# Supplementary material for: Mitigation of multi-scale biases in cell-type deconvolution for spatially resolved transcriptomics using HarmoDecon
Source: Bioinformatics. 2025 Aug 11;41(9):btaf451. doi: 10.1093/bioinformatics/btaf451 (PMC12417081; doi:10.1093/bioinformatics/btaf451)
Supplement: btaf451_Supplementary_Data [file btaf451_supplementary_data.pdf]

Supplemental Materials

Mitigation of multi-scale biases in cell-type deconvolution for spatially resolved transcriptomics using HarmoDecon

Wang Zirui[1], Xu Ke[1], Liu Yang[1], Xu Yu[1], Zhang Lu[1*]
1. Department of Computer Science, Hong Kong Baptist University, Kowloon Tong, Hong Kong

Supplemental Notes

The structure of the HarmoDecon model:

HarmoDecon is based on Gaussian Mixture Graph Convolutional Networks (GMGCN). Similar to Gaussian Mixture Variational Autoencoder (GMVAE), our model combines the flexibility of Gaussian mixture models (GMM) with the generative power of variational autoencoders(VAE) to learn complex latent variable representations in high-dimensional data. To enable the model to learn from graph structures, we substitute the dense layers within the encoders with graph convolutional layers, allowing nodes to consider their neighboring nodes in the graph.

Let $A \in R^{n\times n}$ be the adjacency matrix of an unweighted graph (either a spatial graph or a gene expression graph) with node features $X = [x_1, x_2, \dots, x_n] \in R^{n\times g}$, where n is the number of nodes (spots) and g is the number of features (genes). $Z = [z_1, z_2, \dots, z_n] \in R^{n\times 512}$ is embeddings of X in the hidden layer. $C = [c_1, c_2, \dots, c_n] \in R^{n\times h}$ is a set of one-hot vectors to determine the $i - th$ Gaussian distribution to generate z_I , while h is the number of Gaussian Mixture components.

The latent variable generated by the $i - th$ Gaussian component of GMGCN can be formulated $z_i|c_i \sim N(\mu_{c_i}, \sigma_{c_i}^2 I)$, with means $\mu_{c_i}$and variances $\sigma_{c_i}^2$. The one-hot vector $c \sim Cate(\pi)$ is sampled from the mixing probability π, which chooses one component from the Gaussian mixture.

The posterior distribution of the inference model is symbolized as $q(z_i|X, A) = N(z_i; \tilde{\mu_i}, \tilde{\sigma_i}^2 I)$, $q(c_i|X, A) \approx p(z_i) = \frac{\pi_{c_i} p(c_i')}{\sum_{c_i'=1}^{m} \pi_{c_i'} p(c_i')}$, $q(z_i, c_i|X, A) = q(z_i|X, A)q(c_i|X, A)$.

The parameters of Gaussian distribution $[\tilde{\mu}, log\tilde{\sigma}^2]$ are generated by using a two-layer graph convolutional network (GCN) based encoder.

The latent variable z_i is obtained from $q(z_i|X, A) = N(z_i; \tilde{\mu_i}, \tilde{\sigma}_i^2 I)$ using the reparameterization trick $z_i = \tilde{\mu_i} + \tilde{\sigma_i} \circ \epsilon$, where $\epsilon \sim N(0, I)$. For node i and node j, we suppose the edge between i and j can be represented by Z as $A_{ij} \mid Z \sim Ber\left(sigmoid(z_i^T z_j)\right)$, through which we design the decoder and make the model reconstruct the edges.

For the deconvolution task, z_i is further passed to a two-layer Multilayer Perceptron (MLP), projected to the dimension of cell-type number. We define $f_\theta: z_i \rightarrow \tilde{y_i} \in R^{CT}$, where CT represents the number of cell types.

$$\tilde{y_i} = f_\theta(z_i; W_{\theta_1}, b_{\theta_1}, W_{\theta_2}, b_{\theta_2}) = softmax(W_{\theta_2}(W_{\theta_1} z_i + b_{\theta_1}) + b_{\theta_2})$$

The predicted cell-type vector $\tilde{y_i}$ is used to calculate mean square loss.

To alleviate the platform effect, we deployed an adversarial domain adaptation module. $z_i$is passed to a one-layer MLP $f_\varphi: z_i \rightarrow (0,1)$ called discriminator. The discriminator tries to make the model distinguish the data sources (from scRNA-seq or SRT) and extract distinct features for the two platforms. However, we want the encoder to extract common features of two platforms and project them into an overlapping latent space. To achieve so, we employed a gradient reversal layer before the discriminator:

$$R(z_i) = z_i$$

$$\frac{dR}{dz_i} = -1$$

During backpropagation, the model presents an inverse gradient for the parameters of the encoder, making it more likely to extract common features to trick the discriminator.

The discriminator is formulated as:

$$m_i = f_\varphi(z_i; W_\varphi, b) = sigmoid(W_{\varphi z_i} + b)$$

The domain labels m_i are then used to calculate the cross-entropy loss.

Data propagation during inference of the HarmoDecon model

Let $A \in R^{n \times n}$ be the adjacency matrix of an unweighted graph (either a spatial graph or a gene expression graph) with node features $X = [x_1, x_2, \dots, x_n] \in R^{n \times g}$, where n is the number of nodes (spots) and g is the number of features (genes).

Given an SRT data, the spatial graph and gene expression graph are processed in the model independently. The two graphs are represented as different adjacency matrix A, with the same node features X. Here we formulate the input adjacency matrix as $\widehat{A}$, representing either a spatial graph or an expression graph.

The typical data propagation is shown as below:

First, X and $\hat{A}$ are inputted into a two-layer graph convolutional neural network f_{c}.

$$H^{(1)} = \hat{A}XW_{\mathrm{c}}^{(0)} \tag{1}$$

$$H^{(2)} = \hat{A}H^{(1)}WW_{\mathrm{c}}^{(1)} \tag{2}$$

$$H^{(2)} = \left[h_1^{(2)}, h_2^{(2)}, \ldots, h_n^{(2)}\right] \in R^{n\times 512} \tag{3}$$

Then, $H^{(2)}$is used for calculating a set of latent vectors $C = [c_1, c_2, \ldots, c_n] \in R^{n\times h}$, which determines the chosen Gaussian Mixture component. A one-layer Multilayer Perceptron (MLP) $f_p: H^{(2)}, \rightarrow H^{(3)} \in R^{n\times h}$ is used for dimension projection.

$$H^{(3)} = W_p H^{(2)} + b_p \tag{4}$$

$$C = softmax(H^{(3)} + G) \tag{5}$$

Where $G = -\,log(-\,log(U + \epsilon) + \epsilon)$ follows Gumbel distribution, of which $U \sim$ Uniform$(0,1) \in R^{n\times h}$ and ϵ is a small constant to avoid numerical issues.

Note that C is not strictly one-hot encoded due to the use of the Gumbel-Softmax, which makes the value of the selected dimension close to 1. This approach improves time complexity by reducing the number of gradient estimations required.

Then, C is combined with the original input X to calculate the gaussian mixture embedding $Z = [z_1, z_2, \ldots, z_n] \in R^{n\times 512}$.

$$X' = \text{concat}(X, C) \tag{6}$$

Similar to X, X' is further processed by another two-layer graph convolutional neural network f_x.

$$H^{(5)} = \hat{A}X'W_{\mathrm{x}}^{(0)} \tag{7}$$

$$H^{(6)} = \hat{A}H^{(5)}W_{\mathrm{x}}^{(1)} \tag{8}$$

$$H^{(5)} = \left[h_1^{(5)}, h_2^{(5)}, \ldots, h_n^{(5)}\right] \in R^{n\times 512} \tag{9}$$

$H^{(5)}$ is passed to two parallel MLPs, $f_{\tilde{\mu}}: H^{(5)}, \rightarrow \tilde{\mu} \in R^{n\times 512}$ and $f_{\tilde{\sigma}^2}: H^{(5)}, \rightarrow \tilde{\sigma}^2 \in R^{n\times 512}$, to estimate the mean and variance of Gaussian latent variables.

$$\tilde{\mu} = W_{\tilde{\mu}} H^{(5)} + b_{\tilde{\mu}} \tag{10}$$

$$\tilde{\sigma}^2 = W_{\tilde{\sigma}^2} H^{(5)} + b_{\tilde{\sigma}^2} \quad (11)$$

The latent variable z_i is obtained using the reparameterization trick

$$Z = \widetilde{\mu_i} + \widetilde{\sigma_i} \circ \epsilon \quad (12)$$

where $\epsilon \sim N(0, I) \in R^{n \times 512}$.

Z is further passed to a two-layer MLP, projected to the dimension of cell-type number. We define $f_\theta : Z \to \tilde{Y} \in R^{n \times CT}$, where CT represents the number of cell types.

$$\tilde{Y} = f_\theta\left(Z; W_{\theta_1}, b_{\theta_1}, W_{\theta_2}, b_{\theta_2}\right) = softmax\left(W_{\theta_2}\left(W_{\theta_1} Z + b_{\theta_1}\right) + b_{\theta_2}\right) \quad (13)$$

The output $\tilde{Y}$ contains n rows, corresponding to n spots. Each column of Y represents one cell type. The value $\widetilde{y_{ij}}$ in the i-th row and the j-th column represents the cell type proportions of cell type j in the i-th cell.

When inputted $\hat{A}$ is built by spatial coordinates, the model outputs $\tilde{Y}_{spatial}$. While inputting $\hat{A}$ built by similarities of gene expression, the model outputs $\tilde{Y}_{expression}$.

The final result is determined by averaging the proportions generated by both the spatial graph and gene expression graph.

$$\tilde{Y}_{pred(SRT)} = \frac{\tilde{Y}_{spatial(SRT)} + \tilde{Y}_{expression(SRT)}}{2} \quad (14)$$

Data propagation during training of the HarmoDecon model

In the Method, we mentioned that HarmoDecon accepts three kinds of graphs. They are (1) one spatial graph for SRT spots, (2) one gene expression graph for SRT spots and (3) 250 gene expression graphs for pseudo-spots.

During the training process, we first process 250 gene expression graphs from pseudo-spots, going through steps from formula (1) to (13). Unlike the inference stage, the predicted proportions are determined by one graph alone.

$$\tilde{Y}_{pred(pseudo)} = \tilde{Y}_{expression(pseudo)} \quad (15)$$

$\tilde{Y}_{pred(pseudo)} = [\tilde{y}_1, \tilde{y}_2, \dots, \tilde{y}_n] \in R^{n \times CT}$ is used for calculating two supervised loss items L_{MSE} , L_{sample} , and one unsupervised item L_{entropy} (See Method 3.5).

For spatial graph and gene expression graph from SRT spots, their outputs $\tilde{Y}_{pred(SRT,spatial)}$ and $\tilde{Y}_{pred(SRT,expression)}$ are processed independently. They also go through steps from formula (1) to (13). The only difference is they do not calculate L_{MSE} and L_{sample}, and L_{entropy}.

For all three types of graphs, there are two additional unsupervised loss items, derived from latent variable Z. L_{recon} is calculated directly from Z and $\hat{A}$, using latent variables to reconstruct the graph edges. While L_{CE} is designed for alleviating platform effects, employing an extra domain adaptation module.

In this adversarial domain adaptation module. Z is passed to a one-layer MLP $f_{\varphi}: Z = [z_1, z_2, \dots, z_n] \rightarrow (0,1)^n$ called discriminator. The discriminator tries to make the model distinguish the data sources (from scRNA-seq or SRT) and extract distinct features for the two platforms.

However, we want the encoder to extract common features of the two platforms and project them into an overlapping latent space. To achieve so, we employed a gradient reversal layer before the discriminator:

$$R(z_i) = z_i \tag{16}$$

$$\frac{dR}{dz_i} = -1 \tag{17}$$

During backpropagation, the model presents an inverse gradient for the parameters of the encoder, making it more likely to extract common features to trick the discriminator.

The discriminator is formulated as:

$$m_i = f_{\varphi}(z_i; W_{\varphi}, b) = sigmoid(W_{\varphi z_i} + b) \tag{18}$$

The domain labels m_i are then used to calculate L_{CE} (See Method 3.5).

Construction of the adjacent matrix of graphs

The adjacency matrix A for the pseudo-spots graph is constructed based on gene expression similarity between spots. Let $G = (V, E)$ represent our graph where:

$V = x_1, x_2, \dots, x_n$ is the vertex set of pseudo-spots.

$E \subseteq V \times V$ is the edge set connecting similar spots.

The adjacency matrix $A \in 0,1^{n\times n}$ is defined by:

$$A_{ij} = \begin{cases} 1 & if\left(\mathbf{x_i}, \mathbf{x_j}\right) \in \mathbf{E} \\ 0 & \text{otherwise} \end{cases} \tag{19}$$

Edges are determined through the following process:

Compute the cosine similarity matrix S where:

$$\mathrm{S}_{ij} = \frac{x_i \cdot x_j}{|x_i|\left|x_j\right|} \tag{20}$$

For each spot x_i, identify its top-6 nearest neighbors $\mathcal{N}_i$:

$$\mathcal{N}_i = x_j \mid S_{ij} \tag{21}$$

Establish mutual edges:

$$\left(x_i, x_j\right) \in E \Leftrightarrow x_j \in \mathcal{N}_i \wedge x_i \in \mathcal{N}_j \tag{22}$$

This mutual k-nearest neighbor approach (with k=6) ensures robust connectivity while maintaining a sparse graph structure. The cosine similarity metric effectively captures gene expression patterns while being invariant to magnitude differences.

For the gene expression graph of SRT, the process is the same. For the spatial graph, the only difference is that it use the the Euclidean distance of spots' spatial coordinates, instead of cosine similarity to find neighbors.

Calculation and approximation of sample-level cell-type fractions

In Results, we introduce two single-cell resolution SRT datasets to evaluate the performance of deconvolution on the sample level. The "Ground Truth" shown in Figure 4 is calculated by summing all the single cells:

$$y_{\text{sample},1} = \frac{\sum_{i=1}^{n} y_i n_{ci}}{\sum_{i=1}^{n} n_{ci}} \tag{23}$$

n and n_{ci} represent the number of spots and the number of cells in the spot i respectively.

In Methods, the sample-level cell-type fraction of the graph built with pseudo-spots is again calculated for L_{sample}. To simplify it, we calculate the fraction in an approximate way:

$$y_{\text{sample},2} = \frac{\sum_{i=1}^{n} y_i}{n} \tag{24}$$

We notice that $\mathrm{y}_{\text{sample},1} = \mathrm{y}_{\text{sample},2}$ when $\mathrm{n}_{\mathrm{c}1} = \mathrm{n}_{\mathrm{c}2} = \cdots = \mathrm{n}_{\mathrm{cn}}$. That is, assuming each pseudo-spot has the same number of cells. For STARmap and osmFISH, cell-type fractions obtained by these two approaches have only a small difference in values (Supplementary Table 1).

Acquisition of cancer labels of the BRCA dataset

In Figure 6f, we original manual annotations of the BRCA_2 dataset, which figures out the invasive carcinoma region. To acquire the spot-level annotations of cancer regions, we first exploited a well-curated cell-spot alignment result from [10], in which spots with more than one cancer cell are considered as cancer regions. Then, we adjusted the margin and removed isolated spot points manually to make them more similar to Figure 6f. Finally, we got a cancer region mask like Figure 6d, which contains 532 spots considered as cancer regions and 1986 spots as normal regions.

For cancer labels in scRNA-seq dataset, we downloaded scRNA-seq data with matching breast cancer subtypes (BRCA_1: ER+HER2+; BRCA_2: HER2+) and cell types from a human breast cancer atlas [11]. For the ER+HER2+ subtypes in BRCA_1, we referred to https://www.ncbi.nlm.nih.gov/geo/query/acc.cgi?acc=GSE176078, with annotations of normal and cancer epithelial cells in the major cell types provided by the author. While for the BRCA_2, there are only cancer epithelial cells annotated in all three HER2+ subtype samples https://www.ncbi.nlm.nih.gov/geo/query/acc.cgi?acc=GSE176078, and we used

the minor cell types provided by the author for detailed annotations, following the same criterion as [10].

Compared methods

We compared HarmoDecon to 11 cell-type deconvolution methods: (1) SPOTlight (version 1.4.1), (2) SpatialDWLS (integrated in the R package Giotto, version 1.1.2), (3) Redeconve (version 1.1.0), (4) Stereoscope (integrated in the python package scvi, version 0.6.8), (5) RCTD (integrated in the R package spacexr, version 2.2.1), (6) Cell2location (version 0.1.3), (7) CARD (version 1.14), (8) DSTG (original version in the GitHub page, https://github.com/Su-informatics-lab/DSTG), (9) Tangram (version 1.0.4), (10) STdGCN (original version in the GitHub page, https://github.com/luoyuanlab/stdgcn), (11) SPACEL (version 1.1.6).

For each method, we employed default parameters on their GitHub pages and followed the steps as the tutorials suggested.

Evaluation metrics

For STARmap and osmFISH datasets, we applied five metrics to evaluate the performance of cell-type deconvolution tools. The five metrics are all calculated by spots, so we take a spot i as an example:

1. Pearson correlation coefficient (PCC):

$$\rho_{\tilde{y}_i, y_i} = \frac{E\left[\left(\tilde{y}_i - \mu_{\tilde{y}_i}\right)\left(y_i - \mu_{y_i}\right)\right]}{\sigma_{\tilde{y}_i}\sigma_{y_i}}$$

Where:

$$\mu_{y_i} = \frac{1}{n}\sum_{i=1}^{n} y_i \,, \mu_{\tilde{y}i} = \frac{1}{n}\sum_{i=1}^{n} \tilde{y}_i$$

$$\sigma_{y_i} = \sqrt{\frac{1}{n}\sum_{i=1}^{n}\left(y_i - \mu_{y_i}\right)^2} \,, \sigma_{\tilde{y}i} = \sqrt{\frac{1}{n}\sum_{i=1}^{n}\left(\tilde{y}_i - \mu_{\tilde{y}i}\right)^2}_i$$

For a given spot, a higher PCC value represents better cell-type deconvolution performance.

2. Structural similarity index measure(SSIM):

$$SSIM(\tilde{y}_i, y_i) = \frac{2\mu_{\tilde{y}_i}\mu_{y_i} + C_1}{\mu_{\tilde{y}_i}^2 + \mu_{y_i}^2 + C_1} \cdot \frac{2\sigma_{\tilde{y}_i y_i} + C_2}{\sigma_{\tilde{y}_i}^2 + \sigma_{y_i}^2 + C_2}$$

C_1 and C_2 are constants (set to 0.01 and 0.03). Higher SSIM indicates better performance.

3. **Root-mean-square deviation (RMSE):**

$$\mathrm{RMSE}(\widetilde{y}_i, y_i) = \sqrt{\frac{1}{CT}\sum_{\mathrm{j}=1}^{\mathrm{CT}}\left(\widetilde{y_{i,j}} - y_{\mathrm{i,j}}\right)^2}$$

$y_{i,j}$ ($\widetilde{y_{i,j}}$) denotes proportion of cell type j in spot i from ground truth (from prediction). For a given spot, a lower RMSE value represents better cell-type deconvolution performance.

4. **Jensen-Shannon divergence (JSD):**

$$\mathrm{JSD}(\widetilde{y}_i, y_i) = \frac{1}{2}\left(D_{\mathrm{KL}}(\widetilde{y}_i|m) + D_{\mathrm{KL}}(y_i|m)\right)$$

Where:

$$m = \frac{1}{2}(\widetilde{y}_i + y_i)$$

D_{KL} is Kullback-Leibler divergence. The JS divergence measures the difference between the two distributions, with a lower value indicating greater similarity and better performance on cell-type deconvolution.

5. **Accuracy Score (AS):**

$$\mathrm{AS}i = \frac{1}{4}\left(\mathrm{RANK}_{i,\mathrm{PCC}} + \mathrm{RANK}_{i,\mathrm{SSIM}} + \mathrm{RANK}_{i,\mathrm{RMSE}} + \mathrm{RANK}_{i,\mathrm{JS}}\right)$$

Ranks are sorted individually for each metric across 11 methods.

We included only the cell types annotated in the scRNA-seq data and excluded any cell types present in the SRT that were not annotated in the scRNA-seq when calculating these metrics.

For the MOB dataset, we employed Adjusted Rand Index (ARI) and purity to evaluate spatial domain clustering. The ground truth and predicted (by K-Means) spatial domain clustering are denoted as $\mathrm{H} = \{\mathrm{h}_1, \ldots, \mathrm{h}_\mathrm{J}\}$ and $\widetilde{\mathrm{H}} = \{\widetilde{\mathrm{h}_1}, \ldots, \widetilde{\mathrm{h}_\mathrm{K}}\}$, respectively.

1. **Adjusted Rand Index (ARI):**

$$\mathrm{ARI} = \frac{\sum_{\mathrm{jk}}\binom{\mathrm{n_{jk}}}{2} - \left[\sum_{\mathrm{j}}\binom{|\mathrm{h_j}|}{2}\sum_{\mathrm{k}}\binom{|\widetilde{\mathrm{h_k}}|}{2}\right]/\binom{\mathrm{n}}{2}}{\frac{1}{2}\left[\sum_{\mathrm{j}}\binom{|\mathrm{h_j}|}{2} + \sum_{\mathrm{k}}\binom{|\widetilde{\mathrm{h_k}}|}{2}\right] - \left[\sum_{\mathrm{j}}\binom{|\mathrm{h_j}|}{2}\sum_{\mathrm{k}}\binom{|\widetilde{\mathrm{h}}k|}{2}\right]/\binom{n}{2}}$$

h and $\tilde{\mathrm{h}}$ represent benchmark and predicted spatial domain clustering.

n is the total number of spots. a_i and b_j represent the numbers of spots in $\mathrm{h_i}$and $\widetilde{\mathrm{h_J}}$ represents the number of spots in cluster j in the predicted clusters.

2. **Purity:**

$$\text{Purity} = \frac{1}{n}\sum_{k} \max_{j}\left|\widetilde{h_k} \cap h_j\right|$$

$\widetilde{h_k}$represents the cluster k in the predicted spatial domain clustering, and h_j represents the cluster j in the benchmark spatial domain clustering. The intersection of $\widetilde{h_k}$ and h_j represents the number of elements correctly assigned to cluster k in the predicted and j in the true clusters.

Supplemental Table

**osmFISH**	by cells	by spots
L2/3 IT CTX-1	0.156242	0.160234
L4/5 IT CTX	0.233447	0.190375
L5 IT CTX	0.04737	0.052223
L5 NP CTX	0.051034	0.053651
L5 PT CTX	0.021722	0.023797
L6 CT CTX	0.121958	0.09716
L6 IT CTX	0.081654	0.07115
L6b CTX	0.128239	0.157939
Lamp5	0.03821	0.045849
Sst	0.057315	0.06644
Vip	0.062811	0.081182

**STARmap**	by cells	by spots
Astro	0.114336	0.119129
Endo	0.107941	0.108231
Excitatory L2/3	0.178712	0.179833
Excitatory L4	0.107702	0.104822
Excitatory L5	0.076246	0.074693
Excitatory L6	0.184083	0.179567
Inhibitory Pvalb	0.03124	0.03172
Inhibitory Sst	0.025254	0.025475
Inhibitory Vip	0.012116	0.01416
Micro	0.014047	0.01416
Olig	0.13596	0.135964
Smc	0.012363	0.012246

Supplementary Table 1 The sample-level cell-type fraction of STARmap[1] and osmFISH[3], calculated by counting single cells and by averaging spots. Ambiguous cell types(e.g. Others) are removed.

STARmap	Time cost (Second)	CPU Memory Usage (MiB)	GPU Memory Usage (If any, MiB)
HarmoDecon	216.59	2670.09	520
Cell2Location	2345.68	5418.46	5784
SPACEL	1531.47	3025.78	7856
Stereoseq	258.01	3748.33	386
Tangram	7.16	4343.25	0
DSTG	210.70	575.17	0
STdGCN	839.40	55820.33	7812
CARD	9.59	5207.02	0
RCTD	98.53	6595.2	0
Redeconve	24.42	7703.4	0
SpatialDWLS	330.01	13895.3	0
Spotlight	308.6	8366.6	0

**osmFISH**	**Time cost (Second)**	**CPU Memory Usage (MiB)**	**GPU Memory Usage (If any, MiB)**
HarmoDecon	288.08	3058.23	442
Cell2Location	1997.56	2382.65	410
SPACEL	1393.40	7960.80	4848
Stereoseq	415.06	2310.30	368
Tangram	8.41	606.88	0
DSTG	96.57	438.69	0
STdGCN	203.69	39263.26	7676
CARD	3.19	1340.8	0
RCTD	37.78	1557.1	0
Redeconve	10.70	3042.3	0
SpatialDWLS	38.29	2026.9	0
Spotlight	35.83	3628.1	0

Supplementary Table 2 The runtime and memory consumption of HarmoDecon and Compared methods in the STARmap[1] and osmFISH data[3].

Supplemental Figures

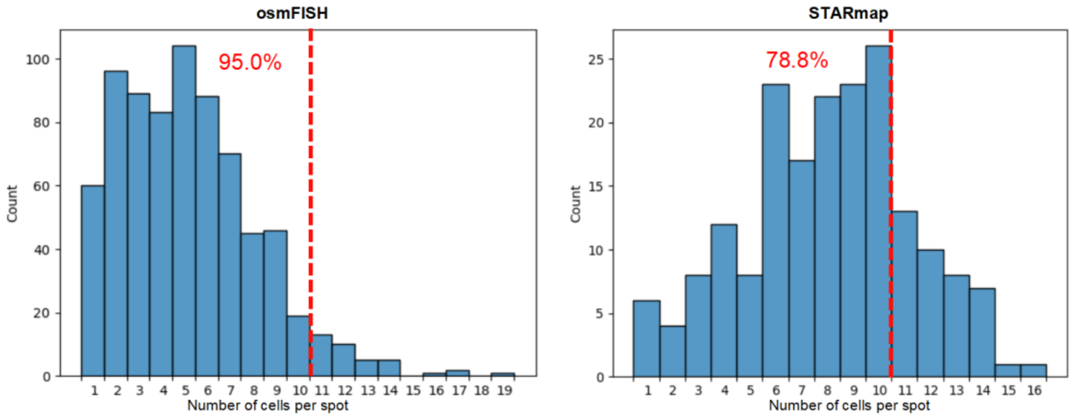

Supplementary Figure 1. Histogram of number of cells per spot in simulated multi-spots datasets. Most simulated multi-cell spots contain 1 to 10 cells. STARmap generally has a larger number of cells per spot than osmFISH.

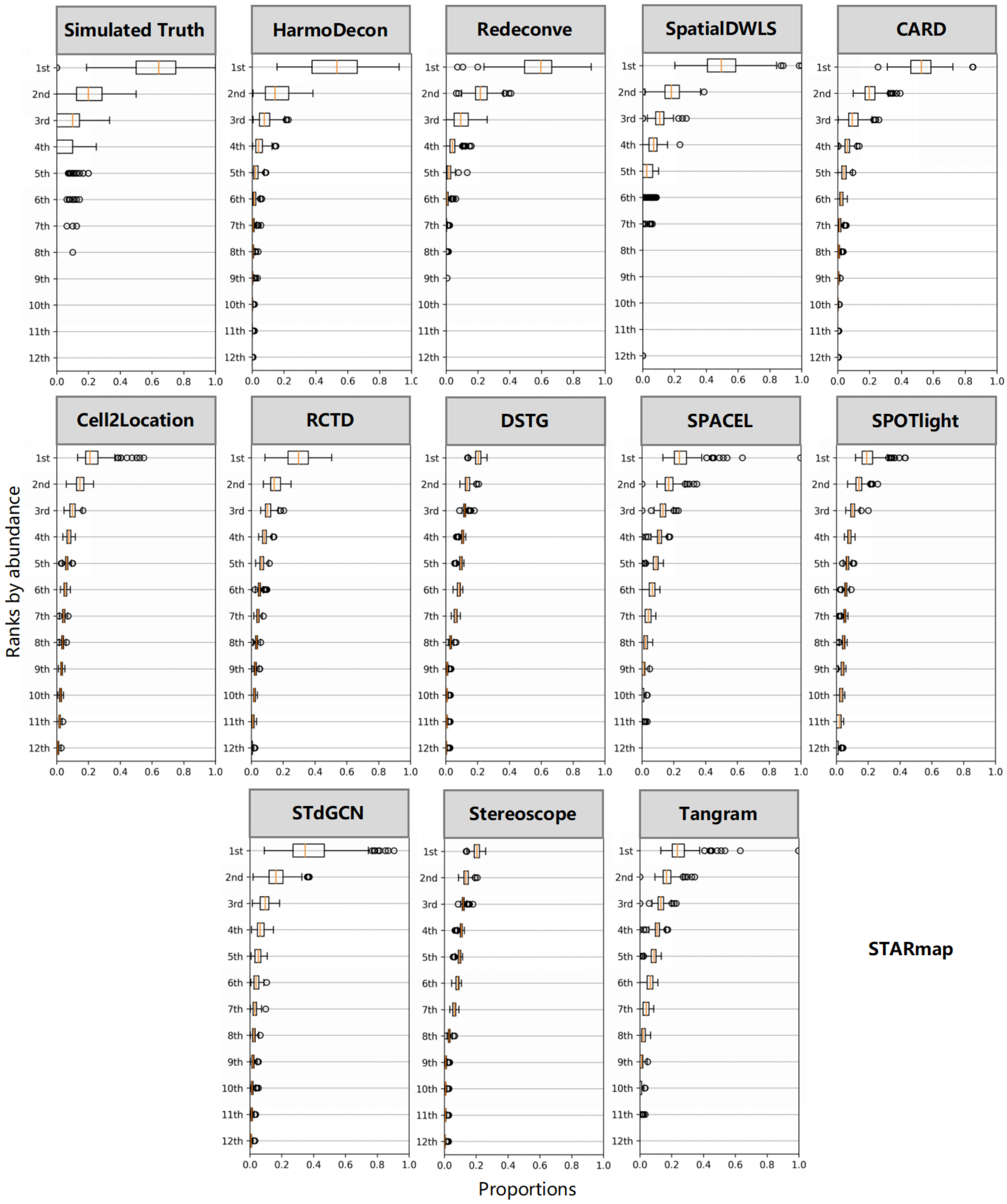
Simulated Truth
HarmoDecon
Redeconve
SpatialDWLS
CARD
Cell2Location
RCTD
DSTG
SPACEL
SPOTlight
STdGCN
Stereoscope
Tangram
STARmap
Ranks by abundance
Proportions
1st
2nd
3rd
4th
5th
6th
7th
8th
9th
10th
11th
12th
0.0
0.2
0.4
0.6
0.8
1.0

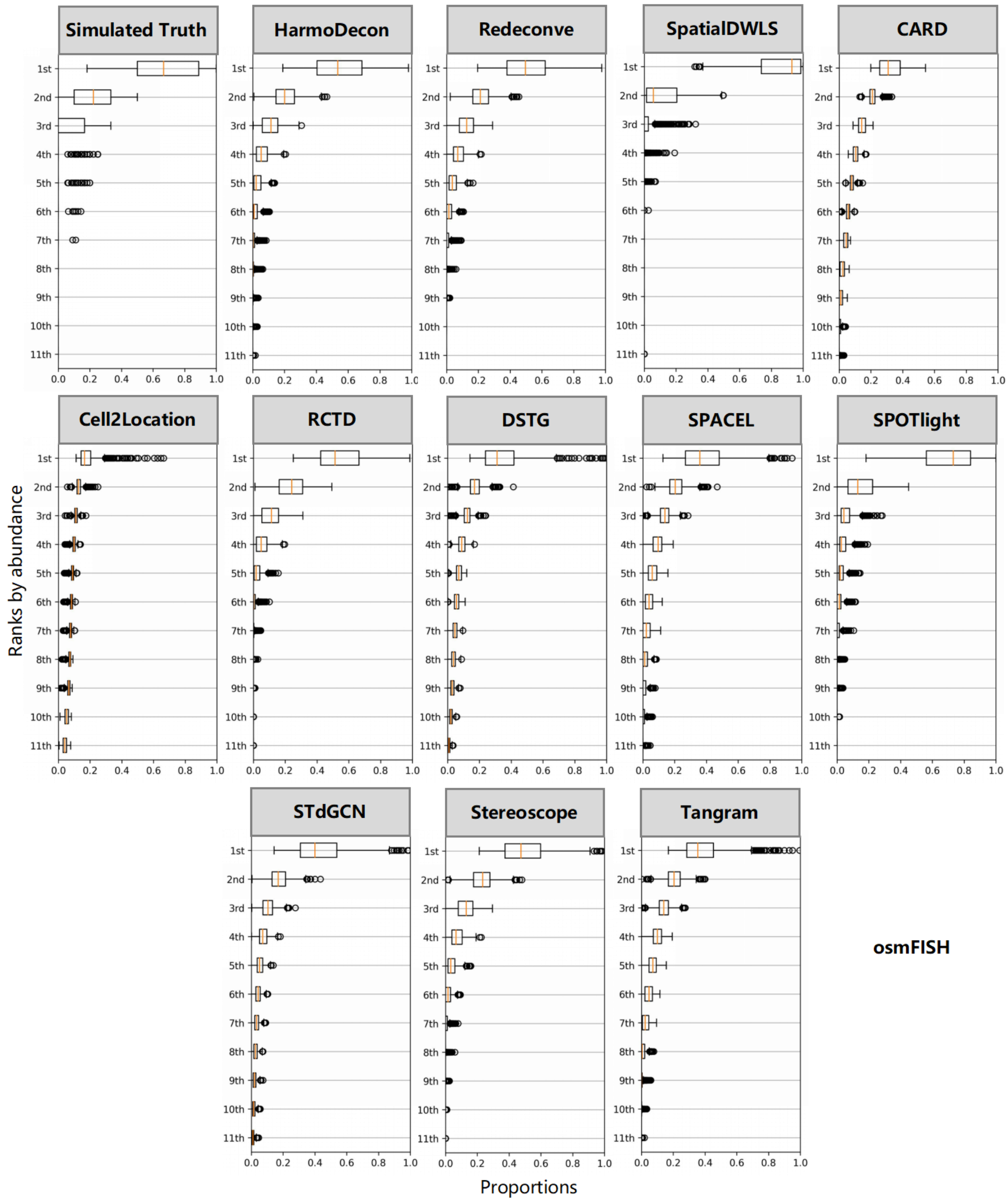

Supplementary Figure 2. The box plot of cell-type proportions ordered by abundance per spot (n = 189) in the simulated STARmap[1] and osmFISH data[3]. We obtained the simulated 'multi-cell spots' STARmap and osmFISH data with cell-type annotations from previous studies[2,4]. We sorted the proportions in each simulated multi-cell spot of the observed truth and results inferred by 12 deconvolution methods. From the most significant proportion values to the last, we displayed their distributions by boxplots.

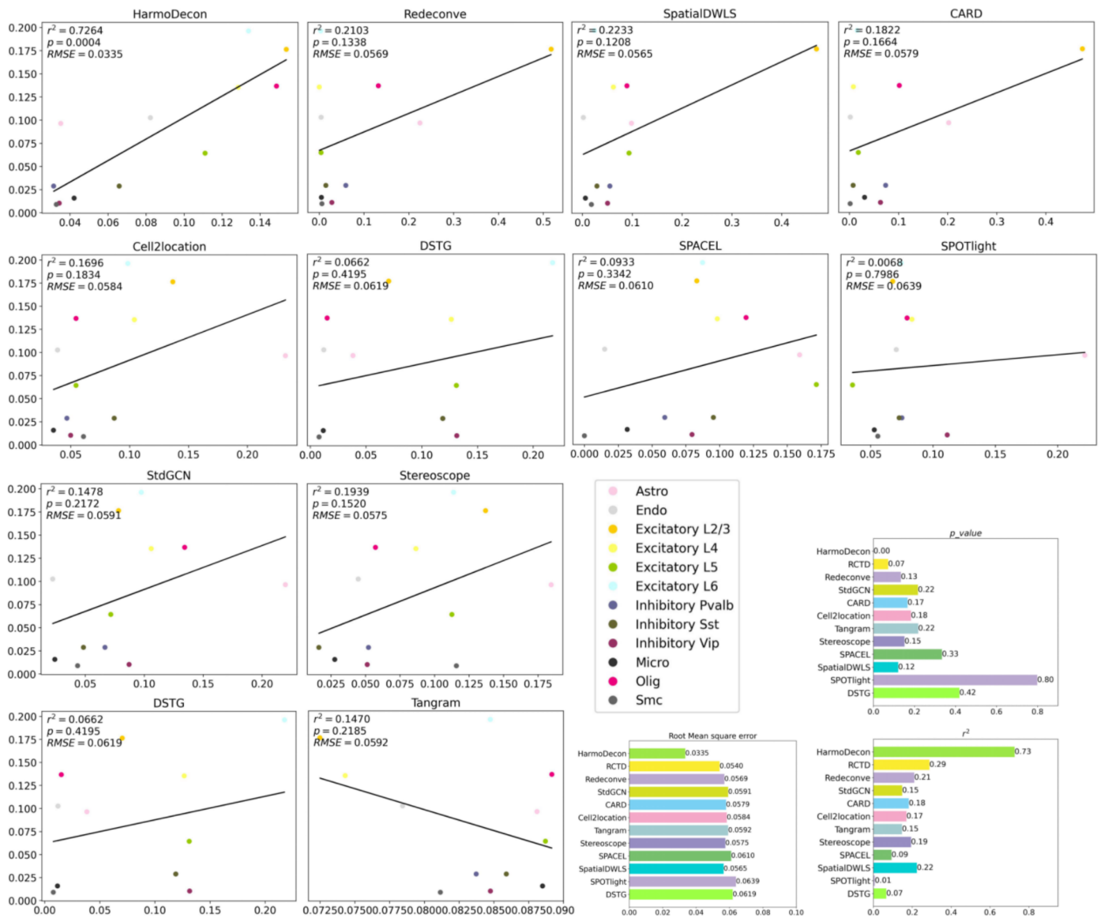
HarmoDecon
$r^2 = 0.7264$
$p = 0.0004$
$RMSE = 0.0335$
Redeconve
$r^2 = 0.2103$
$p = 0.1338$
$RMSE = 0.0569$
SpatialDWLS
$r^2 = 0.2233$
$p = 0.1208$
$RMSE = 0.0565$
CARD
$r^2 = 0.1822$
$p = 0.1664$
$RMSE = 0.0579$
Cell2location
$r^2 = 0.1696$
$p = 0.1834$
$RMSE = 0.0584$
DSTG
$r^2 = 0.0662$
$p = 0.4195$
$RMSE = 0.0619$
SPACEL
$r^2 = 0.0933$
$p = 0.3342$
$RMSE = 0.0610$
SPOTlight
$r^2 = 0.0068$
$p = 0.7986$
$RMSE = 0.0639$
StdGCN
$r^2 = 0.1478$
$p = 0.2172$
$RMSE = 0.0591$
Stereoscope
$r^2 = 0.1939$
$p = 0.1520$
$RMSE = 0.0575$
DSTG
$r^2 = 0.0662$
$p = 0.4195$
$RMSE = 0.0619$
Tangram
$r^2 = 0.1470$
$p = 0.2185$
$RMSE = 0.0592$
Astro
Endo
Excitatory L2/3
Excitatory L4
Excitatory L5
Excitatory L6
Inhibitory Pvalb
Inhibitory Sst
Inhibitory Vip
Micro
Olig
Smc
p_value
HarmoDecon 0.00
RCTD 0.07
Redeconve 0.13
StdGCN 0.22
CARD 0.17
Cell2location 0.18
Tangram 0.22
Stereoscope 0.15
SPACEL 0.33
SpatialDWLS 0.12
SPOTlight 0.80
DSTG 0.42
Root Mean square error
HarmoDecon 0.0335
RCTD 0.0540
Redeconve 0.0569
StdGCN 0.0591
CARD 0.0579
Cell2location 0.0584
Tangram 0.0592
Stereoscope 0.0575
SPACEL 0.0610
SpatialDWLS 0.0565
SPOTlight 0.0639
DSTG 0.0619
r^2
HarmoDecon 0.73
RCTD 0.29
Redeconve 0.21
StdGCN 0.15
CARD 0.18
Cell2location 0.17
Tangram 0.15
Stereoscope 0.19
SPACEL 0.09
SpatialDWLS 0.22
SPOTlight 0.01
DSTG 0.07

STARmap

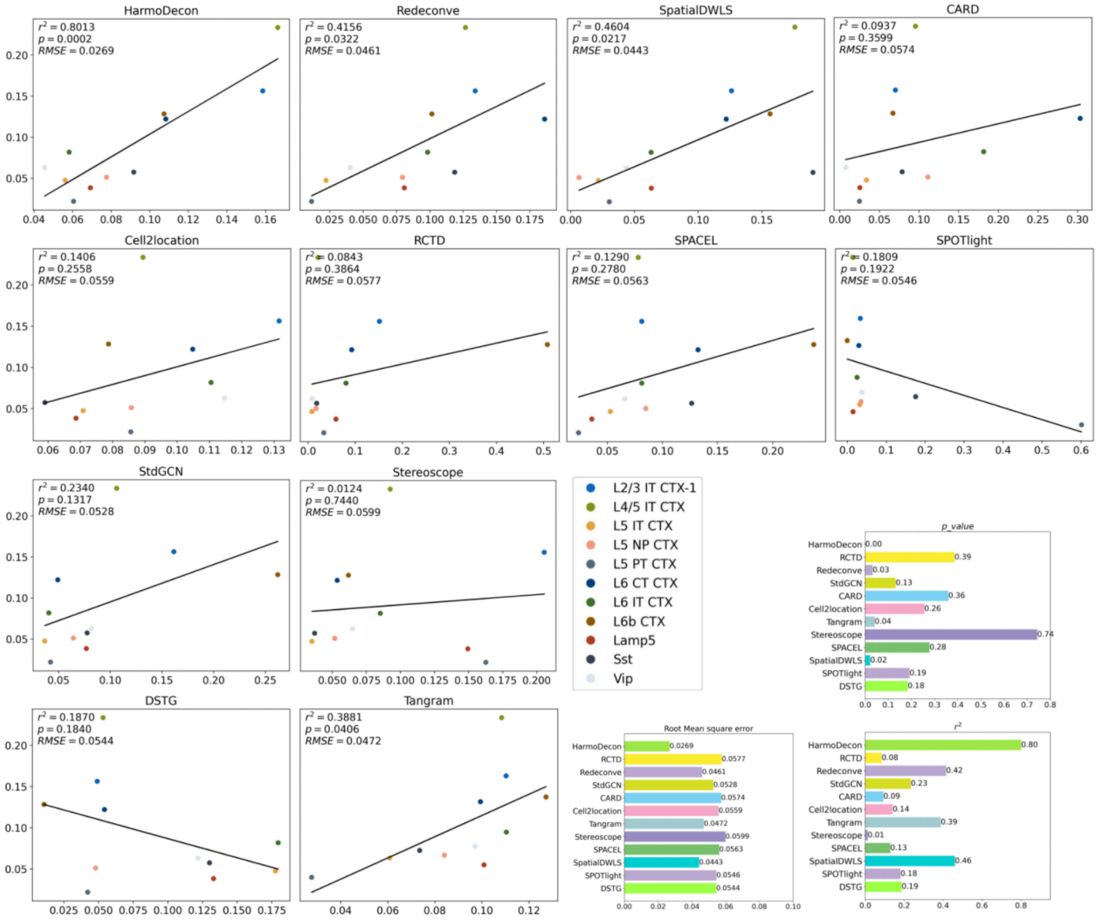

osmFISH

Supplementary Figure 3. HarmoDecon successfully captures the linear correlation when recovering the sample-level cell-type profiles. Apart from demonstrating superior performance in inferring spot-level cell-type proportions, HarmoDecon consistently delivered promising results when summing the proportions of spots to reconstruct sample-level cell-type profiles, showing a high correlation. When examining the p-value for a hypothesis test utilizing the Wald Test with a t-distribution of the test statistic, only HarmoDecon consistently showed significance($p < 0.05$) across the two single-cell spatial transcriptomics datasets. Our method effectively captured the linear relationship in sample cell-type fractions.

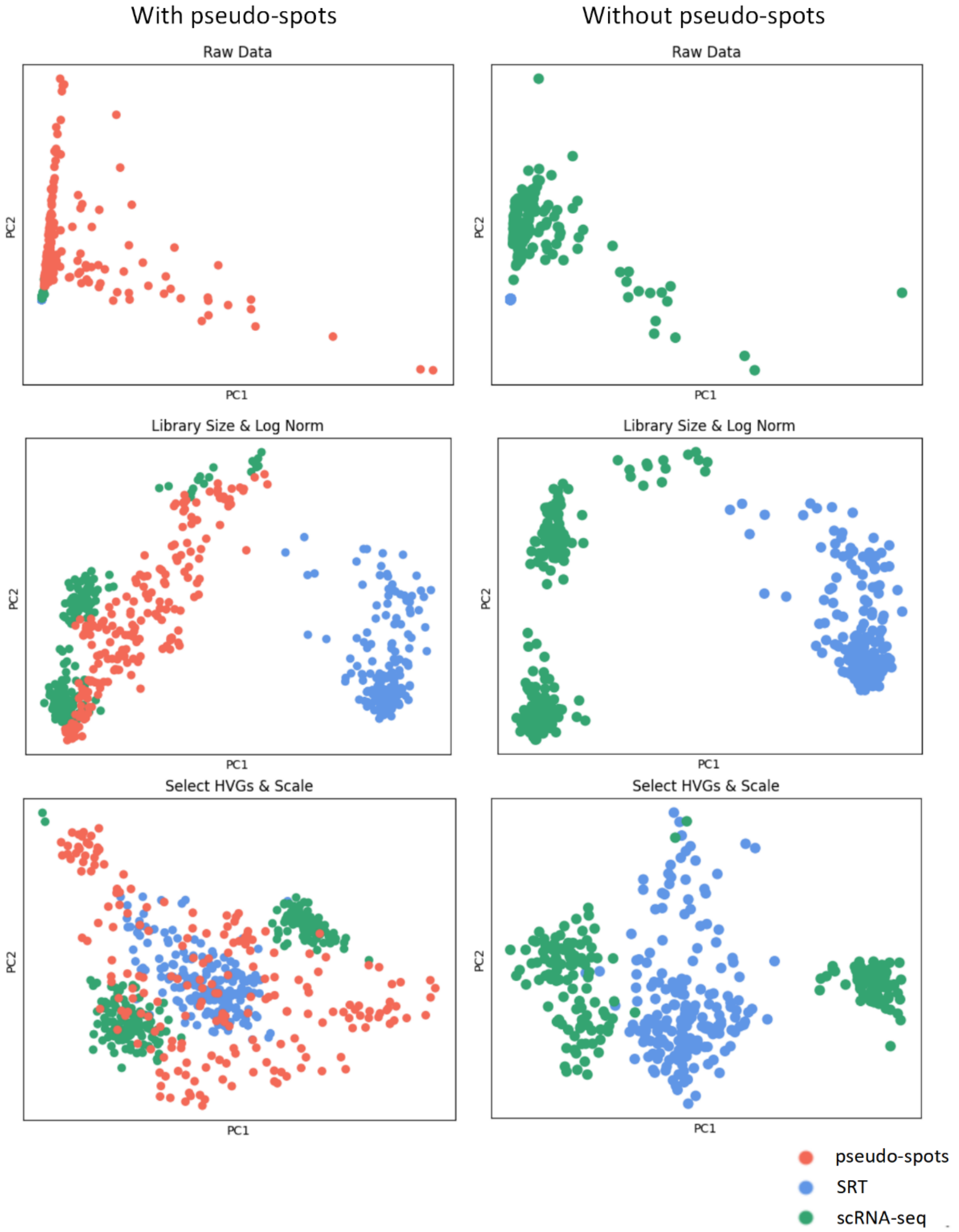

Supplementary Figure 4. Pre-processing can alleviate the platform effects that occur in the STARmap dataset. By looking at the scatter plots after PCA, we observed that the raw gene expression of scRNA-seq (green points) and SRT simulated multi-cell spots (blue points) has a strong deviation. After library size and logarithmic normalization, the raw gene expression of scRNA-seq and SRT points still do not have an overlapping area. As mentioned in the paper, our method generated pseudo-spots (red points) by aggregating the expression values of scRNA-seq. The synthesized pseudo-spots have an overlapping area with the target SRT data used for deconvolution.

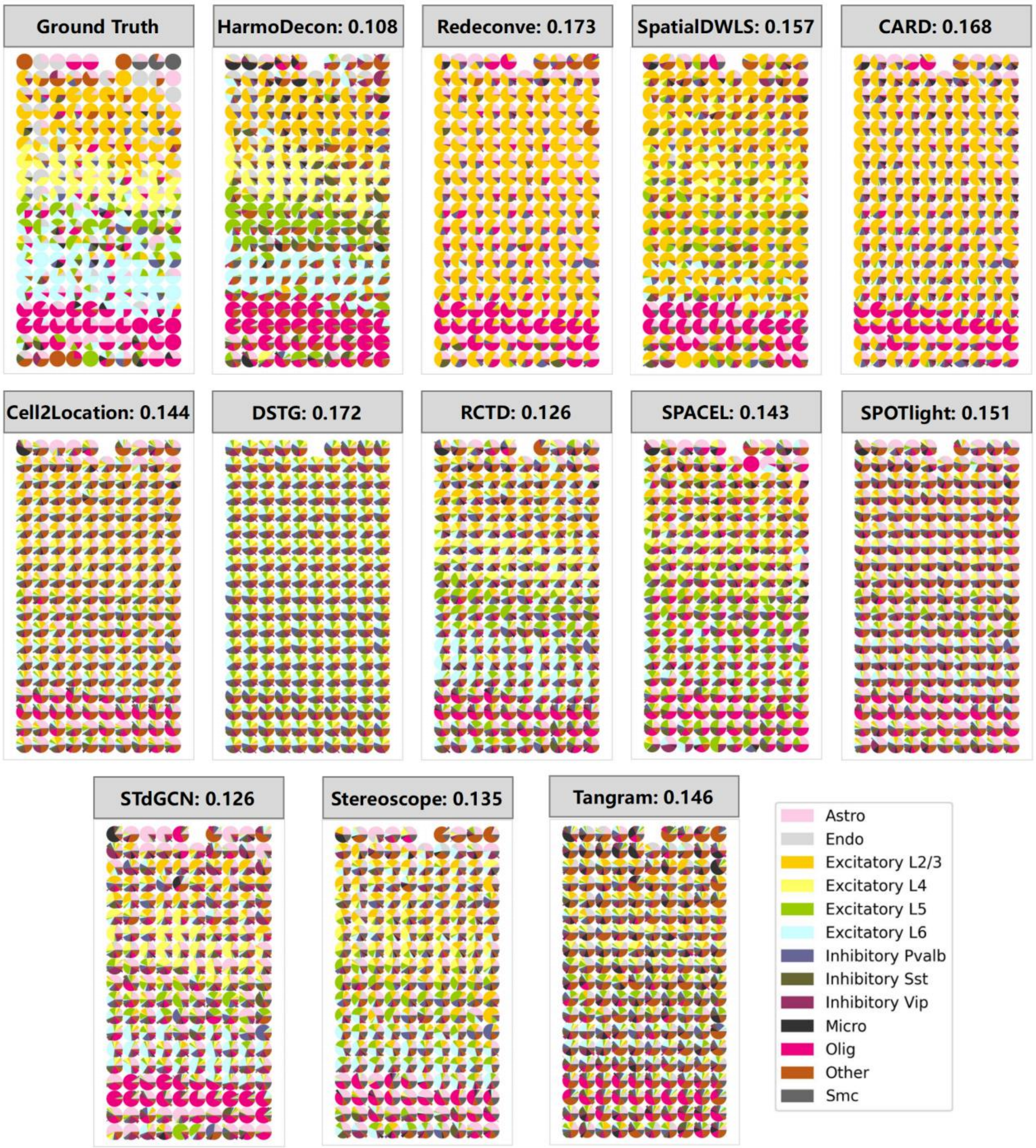

Supplementary Figure 5. Pie charts of the deconvolution results of STARmap mouse visual cortex data. While the article only presents a subset of the results from selected well-performing methods, we have included the complete results here as a supplement. Our observations indicated that HarmoDecon consistently outperforms other methods in terms of the evaluated Root Mean Square Error (RMSE).

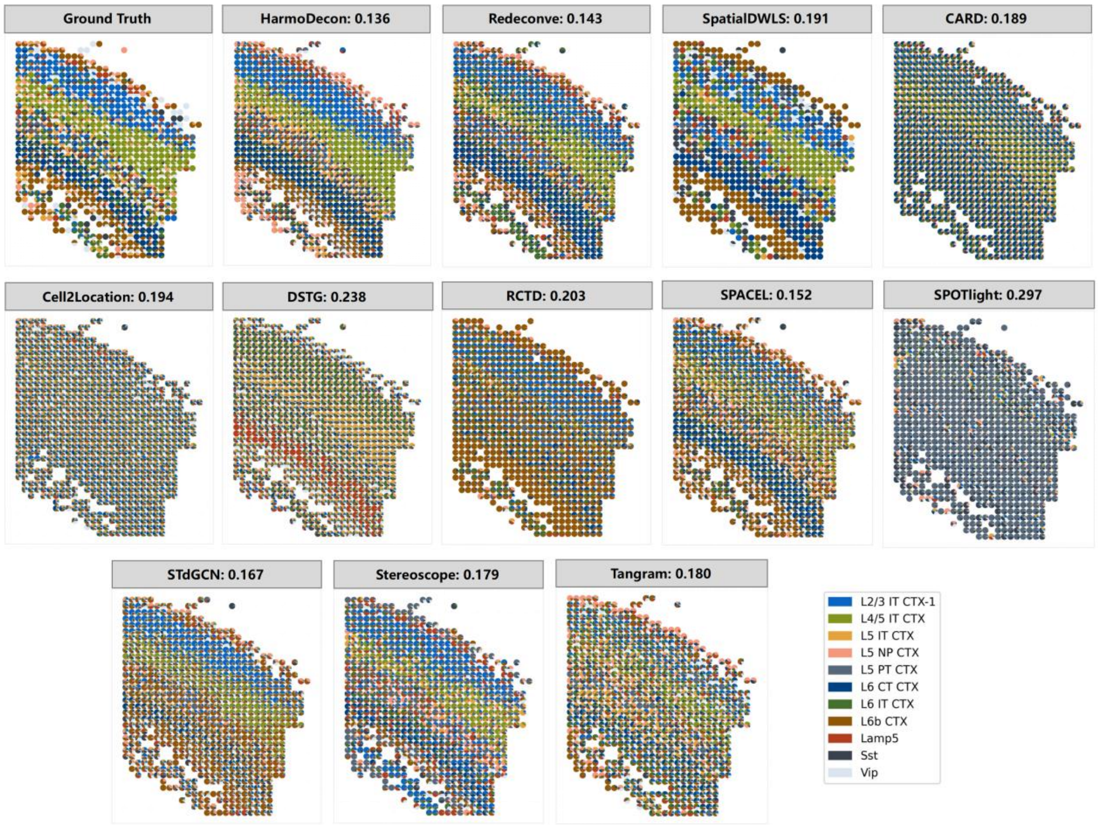

Supplementary Figure 6. Pie charts of the deconvolution results of osmFISH mouse somatosensory data. While the article only presents a subset of the results from selected well-performing methods, we have included the complete results here as a supplement. Our observations indicated that HarmoDecon consistently outperforms other methods in terms of the evaluated Root Mean Square Error (RMSE).

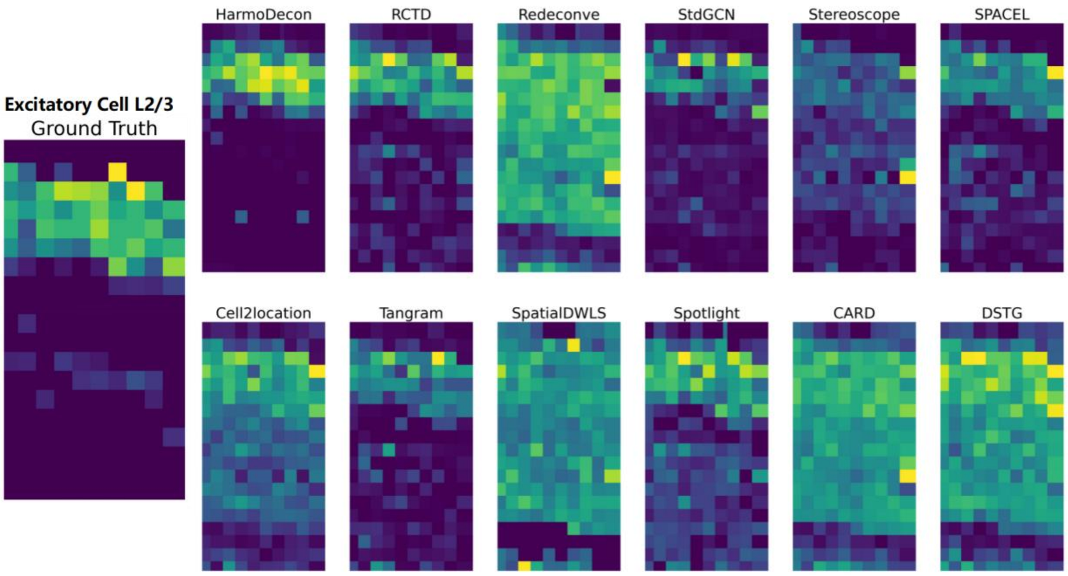
Excitatory Cell L2/3
Ground Truth
HarmoDecon
RCTD
Redeconve
StdGCN
Stereoscope
SPACEL
Cell2location
Tangram
SpatialDWLS
Spotlight
CARD
DSTG

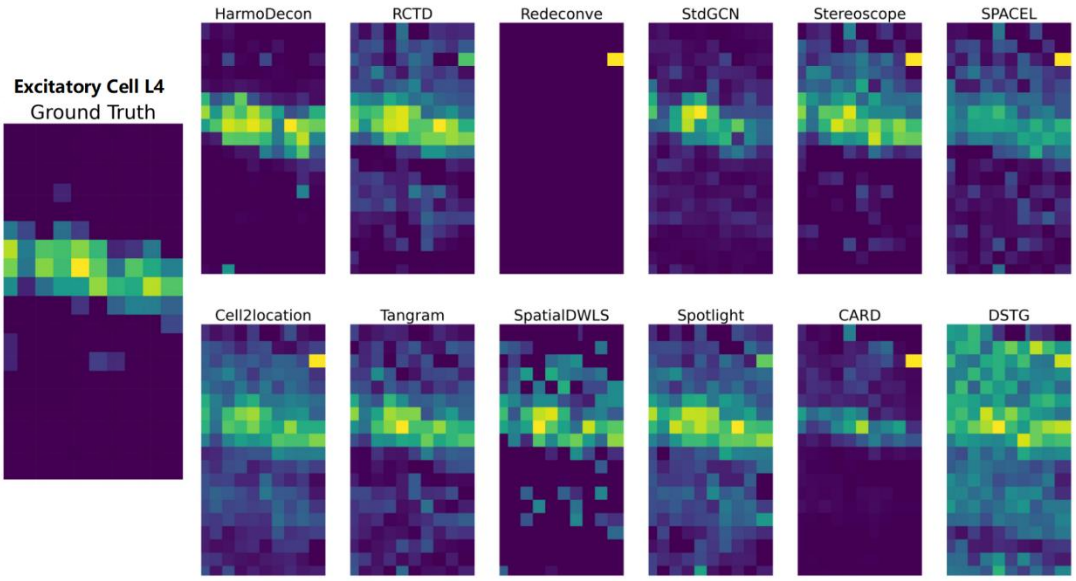
Excitatory Cell L4
Ground Truth
HarmoDecon
RCTD
Redeconve
StdGCN
Stereoscope
SPACEL
Cell2location
Tangram
SpatialDWLS
Spotlight
CARD
DSTG

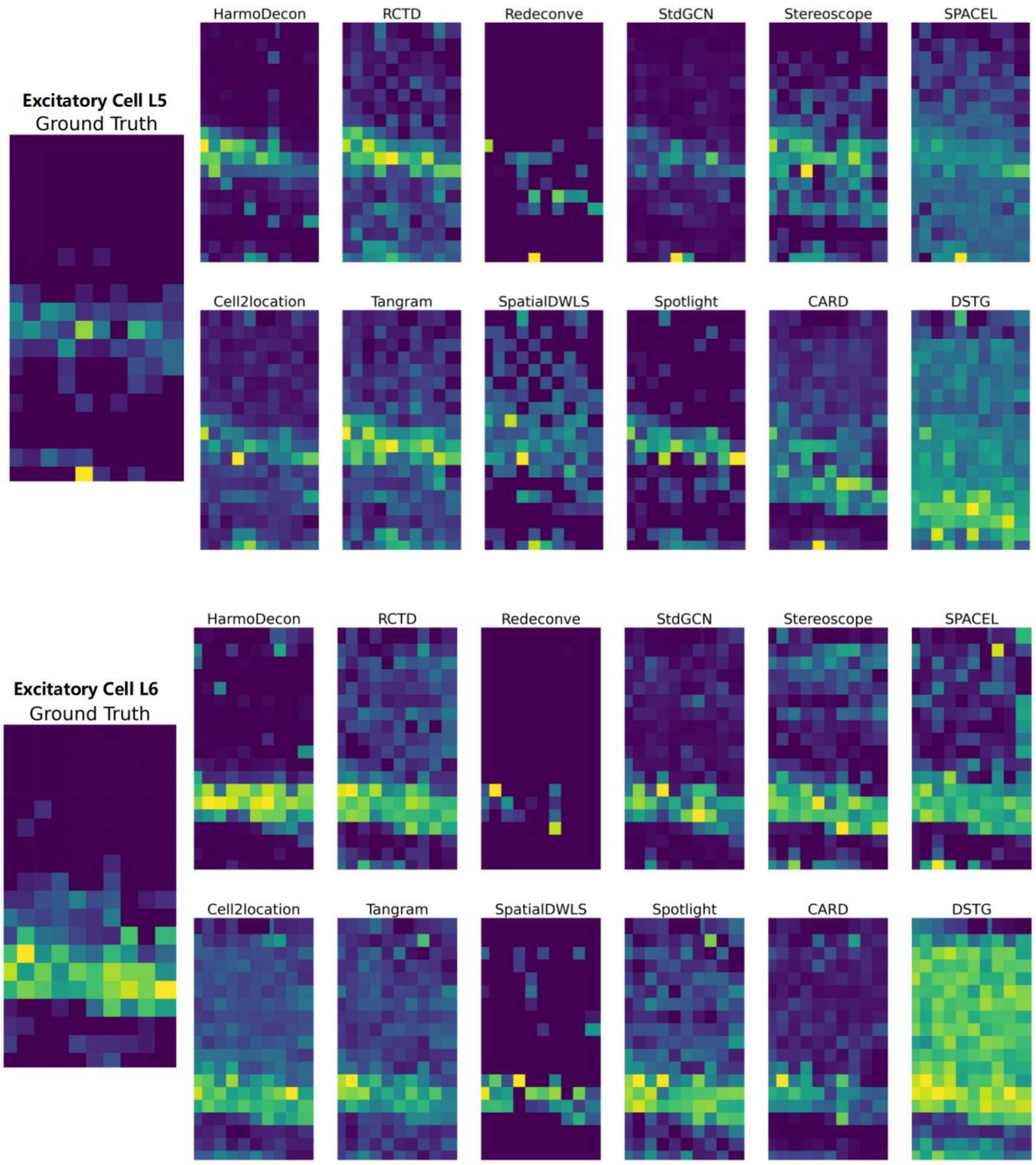

Supplementary Figure 7. Visualization of main cell types in STARmap dataset. We have chosen several layer-specific excitatory cells for demonstration purposes. As illustrated in the figure below, only HarmoDecon, RCTD, and StdGCN consistently exhibited a clear boundary for each anatomical layer. Among these methods, HarmoDecon stood out by having fewer false positive spots compared to the others across the four types of excitatory cells.

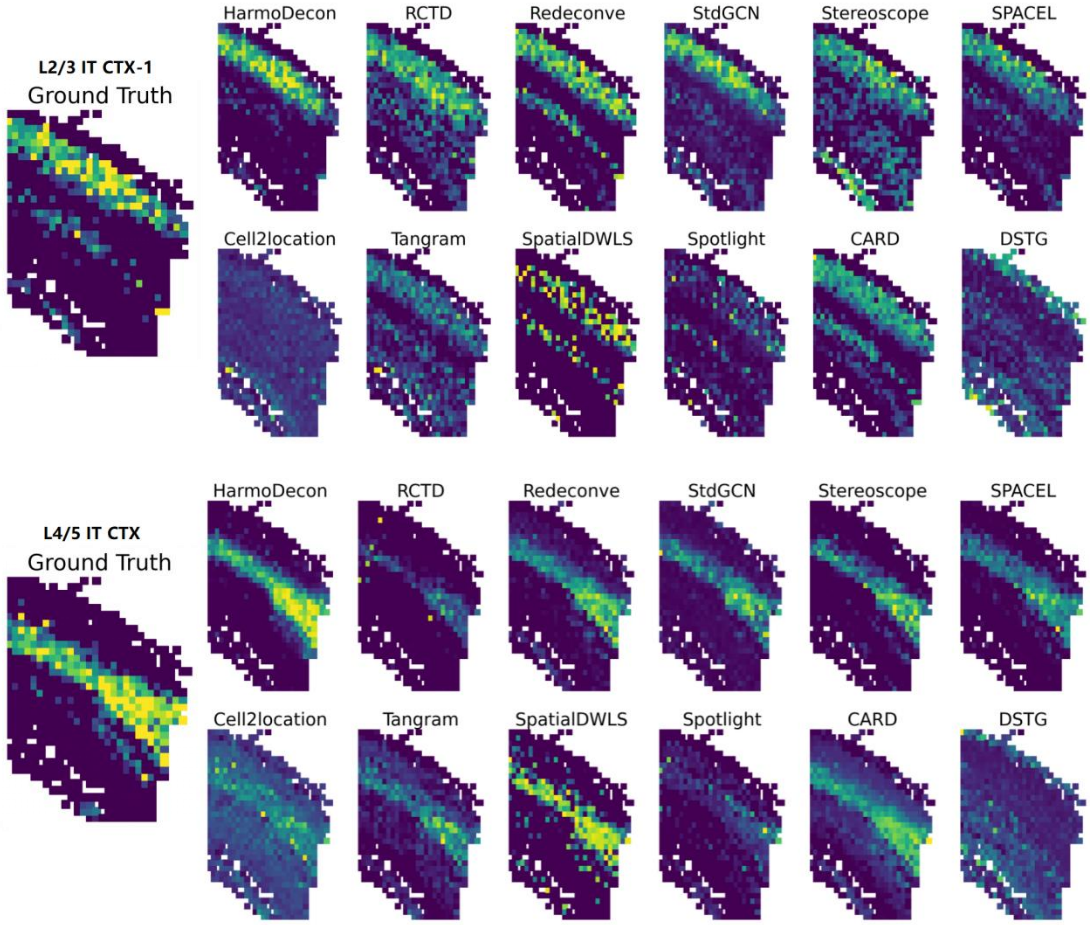
L2/3 IT CTX-1
Ground Truth
HarmoDecon
RCTD
Redeconve
StdGCN
Stereoscope
SPACEL
Cell2location
Tangram
SpatialDWLS
Spotlight
CARD
DSTG
L4/5 IT CTX
Ground Truth
HarmoDecon
RCTD
Redeconve
StdGCN
Stereoscope
SPACEL
Cell2location
Tangram
SpatialDWLS
Spotlight
CARD
DSTG

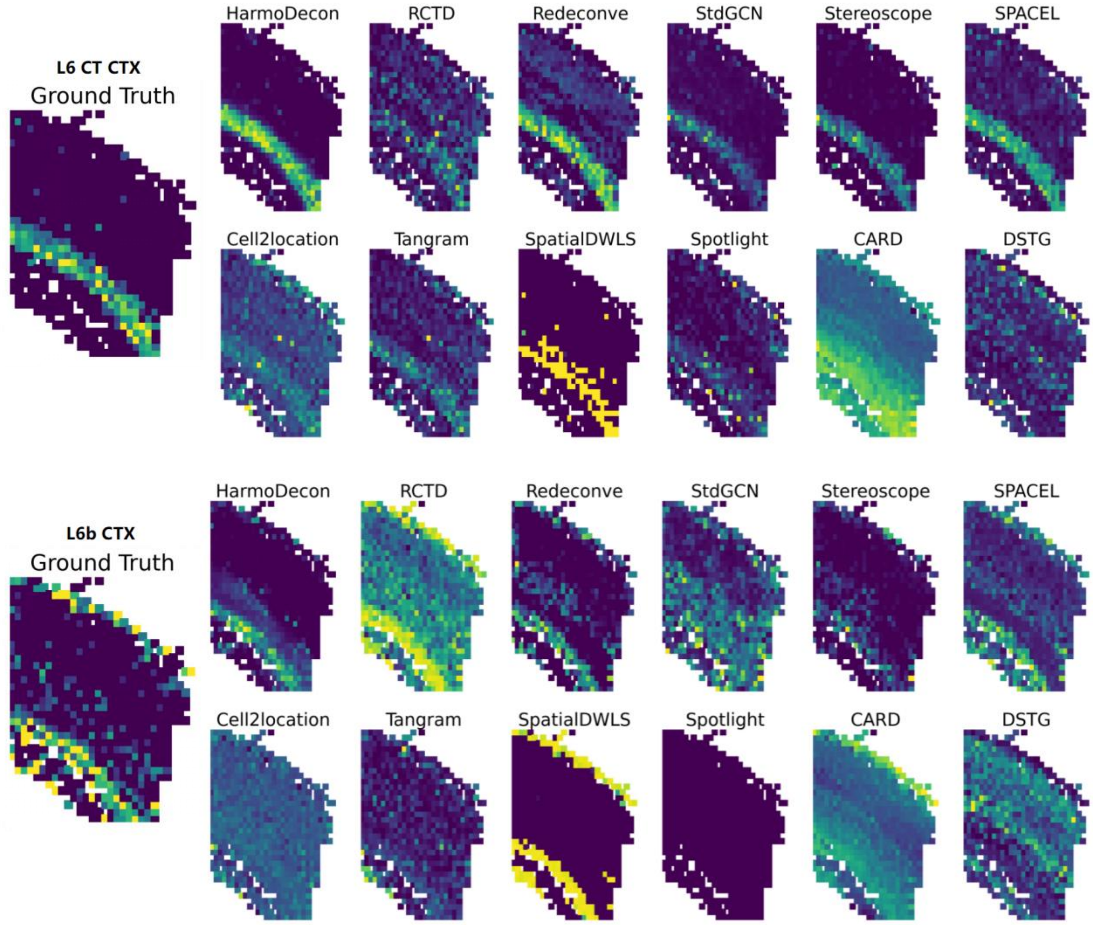

Supplementary Figure 8. Visualization of main cell types in osmFISH dataset. We have chosen several layer-specific excitatory cells for demonstration purposes. As illustrated in the figure below, only HarmoDecon and Redeconve consistently exhibited a clear boundary for each anatomical layer. Among these methods, HarmoDecon stood out by having fewer false positive spots compared to the others across the four types of neuron cells.

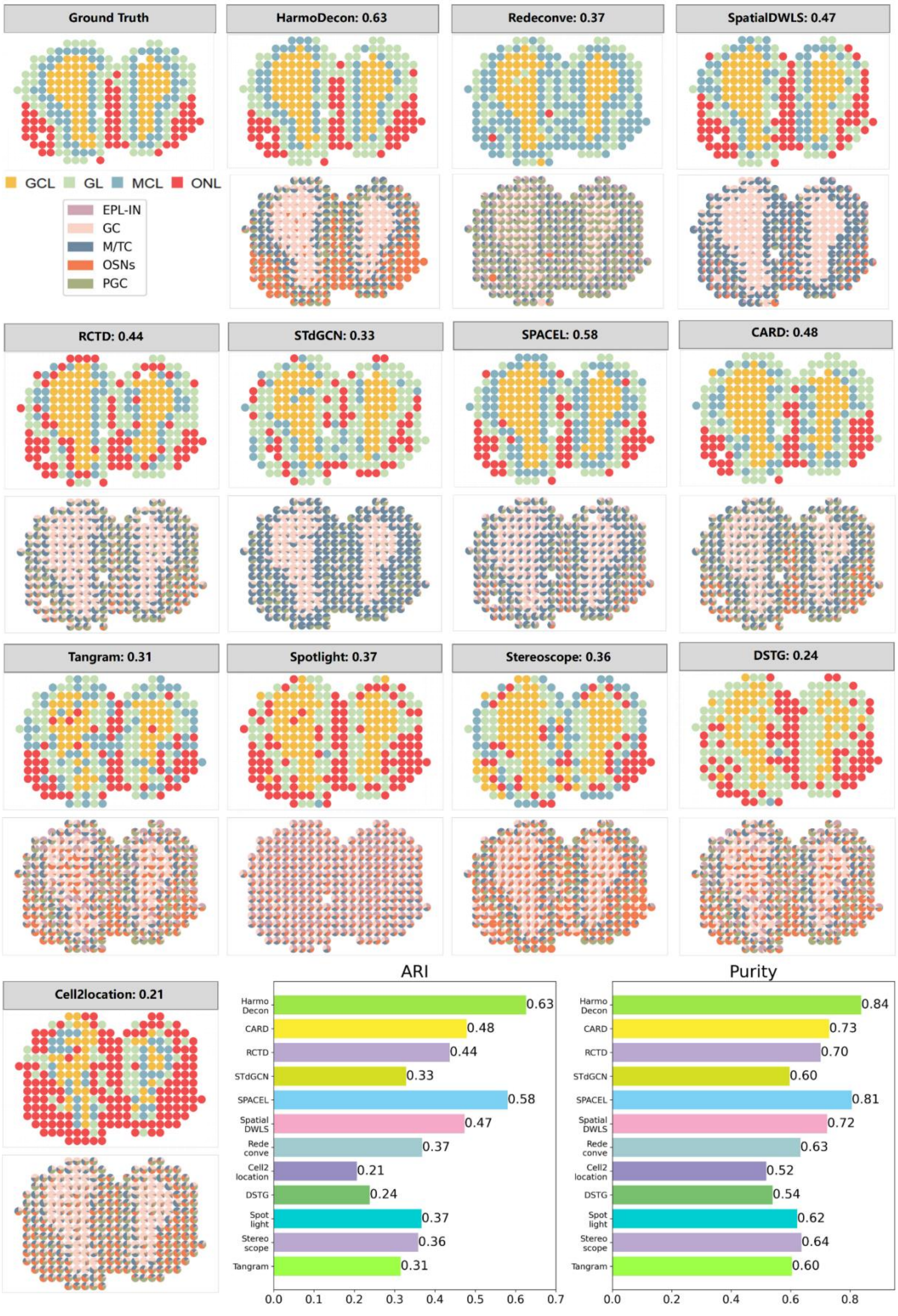

Supplementary Figure 9. Pie charts and domain clustering of the deconvolution results of mouse olfactory bulb (MOB)[6] data by legacy ST. These figures demonstrated that HarmoDecon consistently surpasses other methods in the evaluated Adjusted Rand Index (ARI) and purity with domain clustering by cell types.

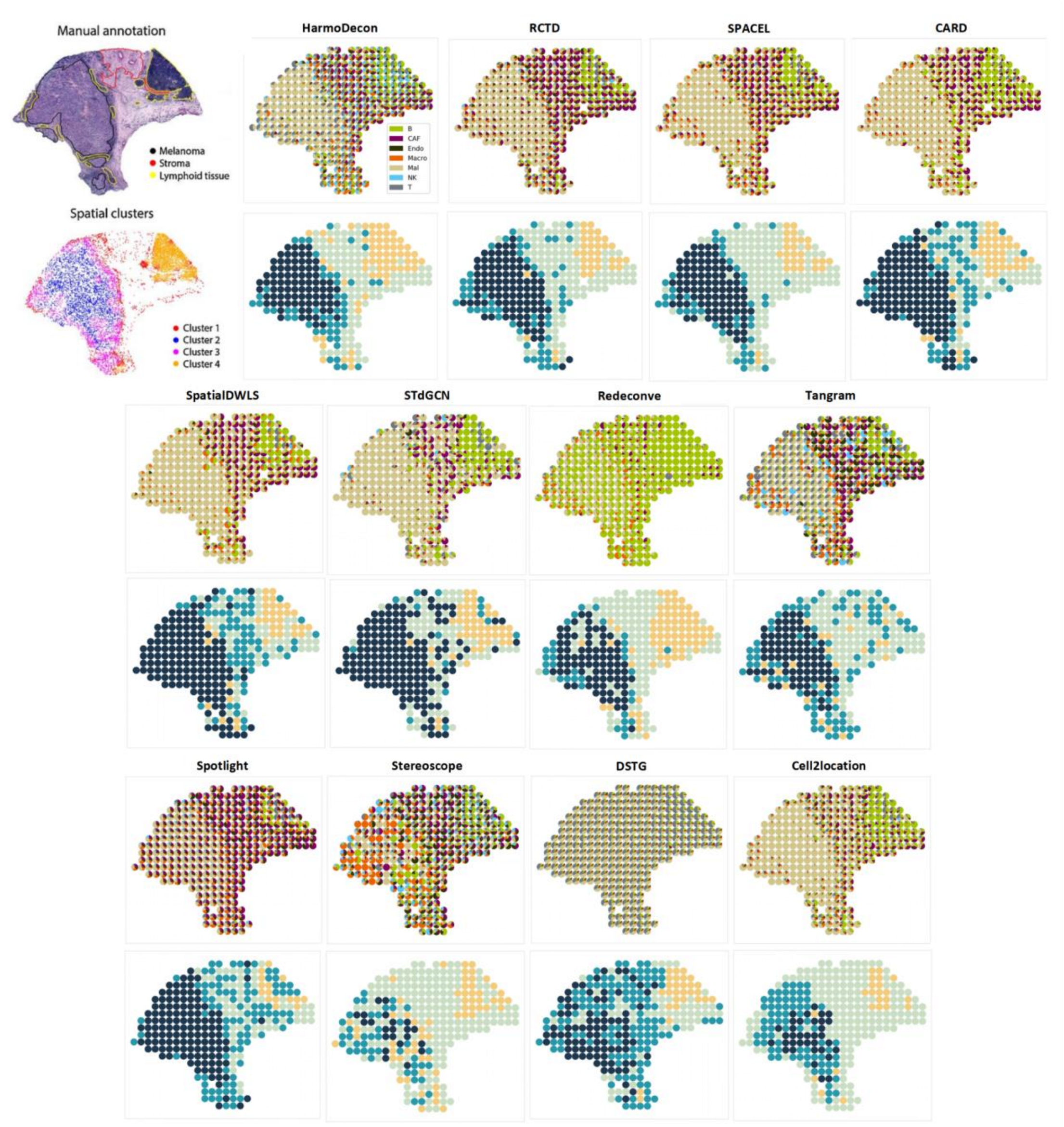

Supplementary Figure 10. Pie chart and domain clustering of the deconvolution results of human melanoma[7] data by legacy ST. Figures show that only HarmoDecon, RCTD, and SPACEL can well distinguish the core melanoma region (dark blue-green) and border melanoma region(light blue-green).

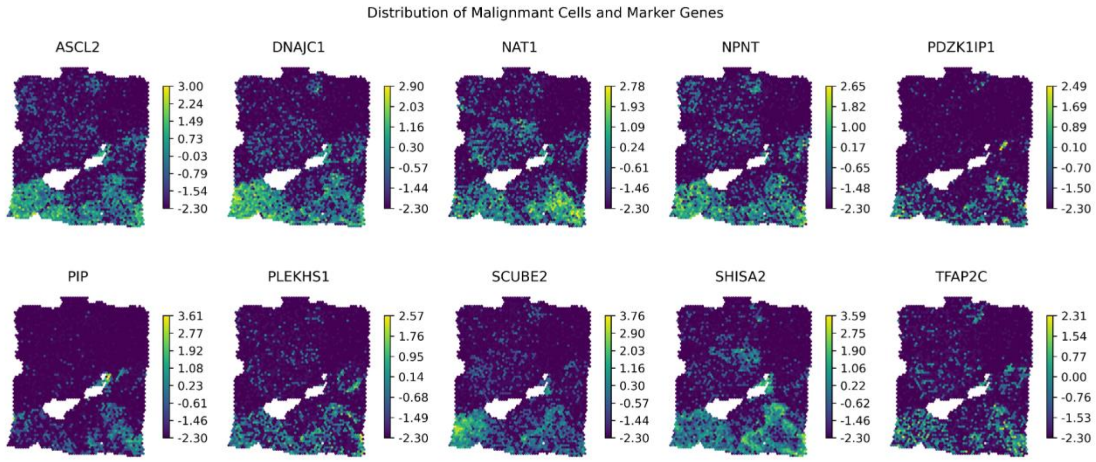

Supplementary Figure 11. Heatmaps of selected 10 marker genes of the BRCA_1 dataset.

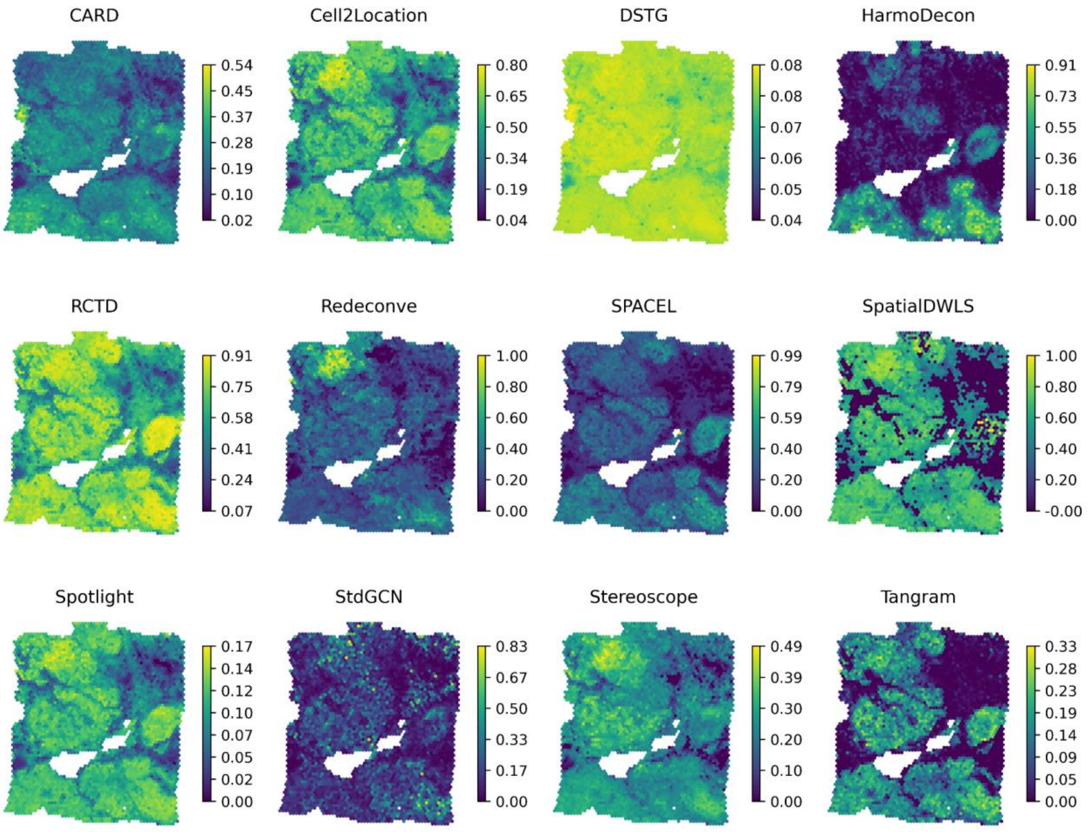

The proportion of cancer epithelial cells inferred by 12 methods

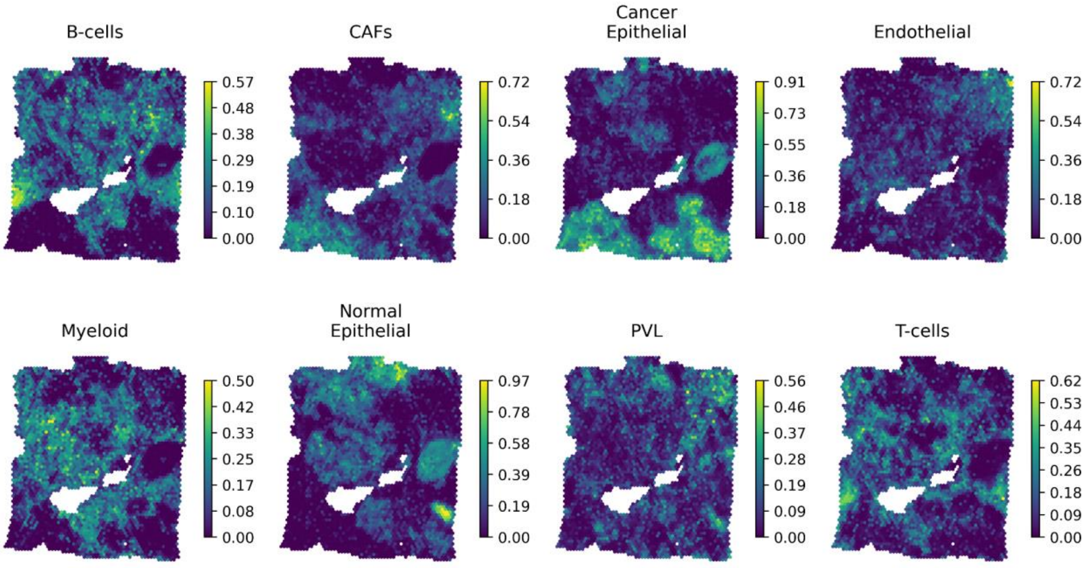

The Proportion of each cell type inferred by HarmoDecon

Supplementary Figure 12. Heatmaps of the proportion of cancer epithelial cells in the BRCA_1 dataset inferred by all 12 methods.

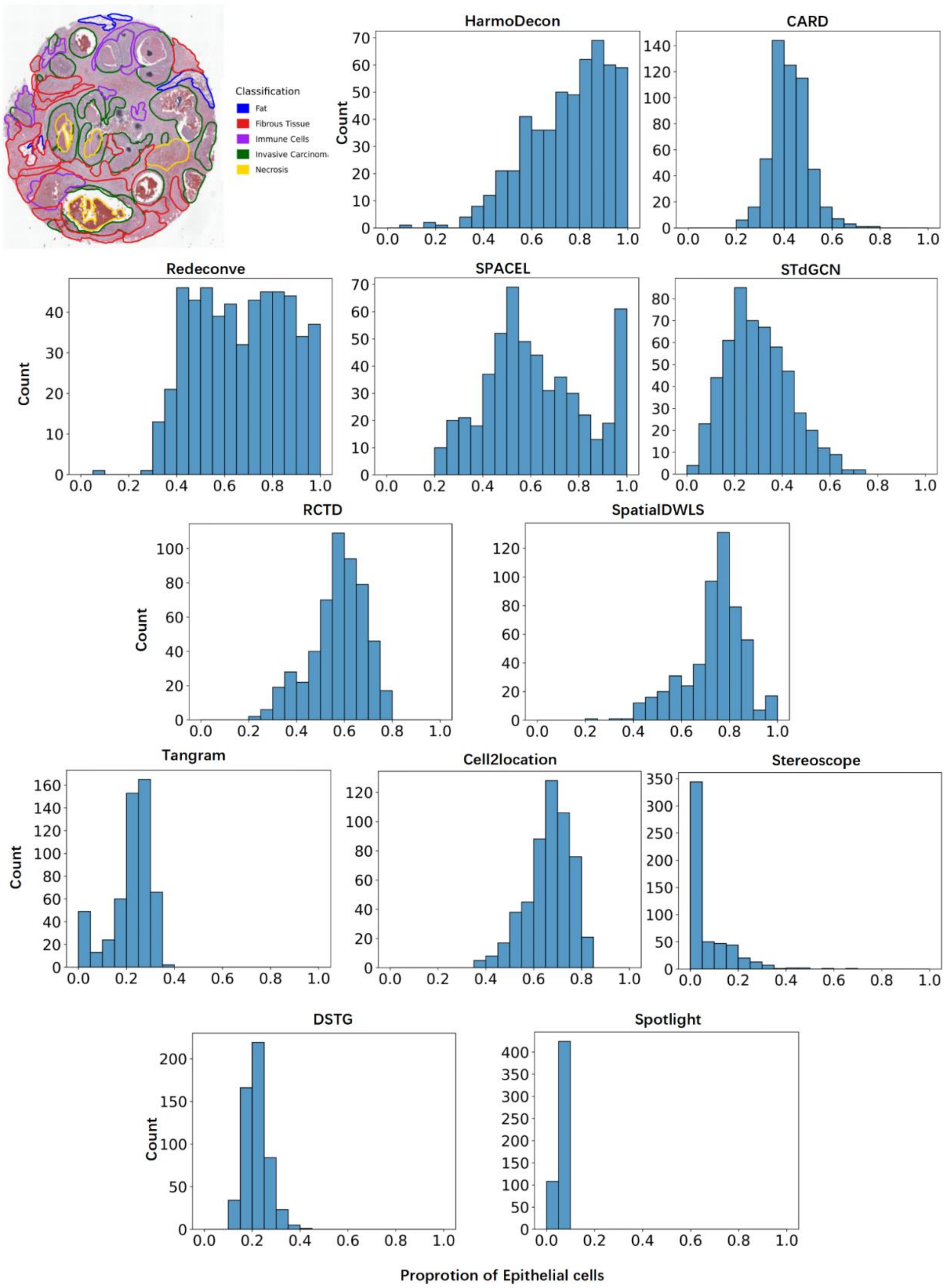

Supplementary Figure 13. HarmoDecon captures the enrichment of epithelial cells in the invasive carcinoma region Breast cancer lesions consist of malignant epithelial cells. In the breast cancer (BRCA_2) dataset generated by 10X Visium[5], we calculated the proportion of epithelial cells within spots located in the invasive carcinoma region (circled by green lines, 532 spots in total). Our observations revealed that among the 7 state-of-the-art methods assessed, HarmoDecon's results notably highlighted the enrichment of epithelial cells. While some modern models like Redeconve and SPACEL also captured this dominance, there were still spots within the carcinoma region that contained a lower concentration of epithelial cells.

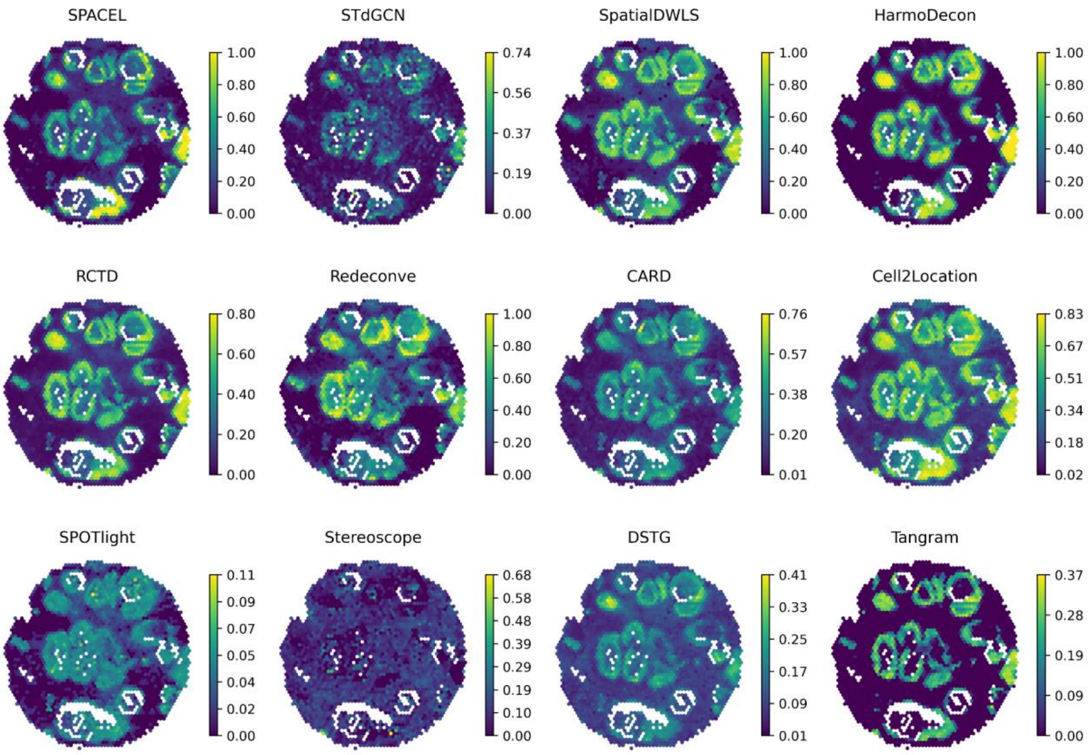

The proportion of cancer epithelial cells inferred by 12 methods

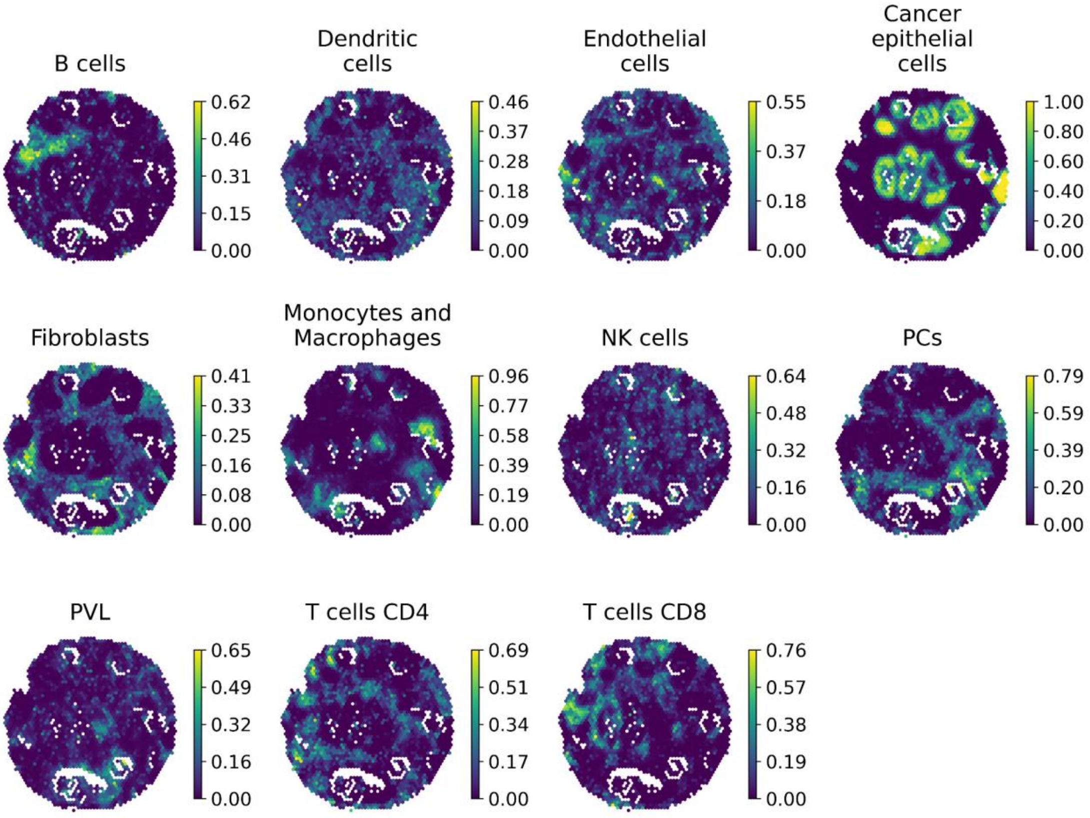

The Proportion of each cell type inferred by HarmoDecon

Supplementary Figure 14. Heatmaps of the proportion of cancer epithelial cells in the BRCA_2 dataset inferred by all 12 methods.

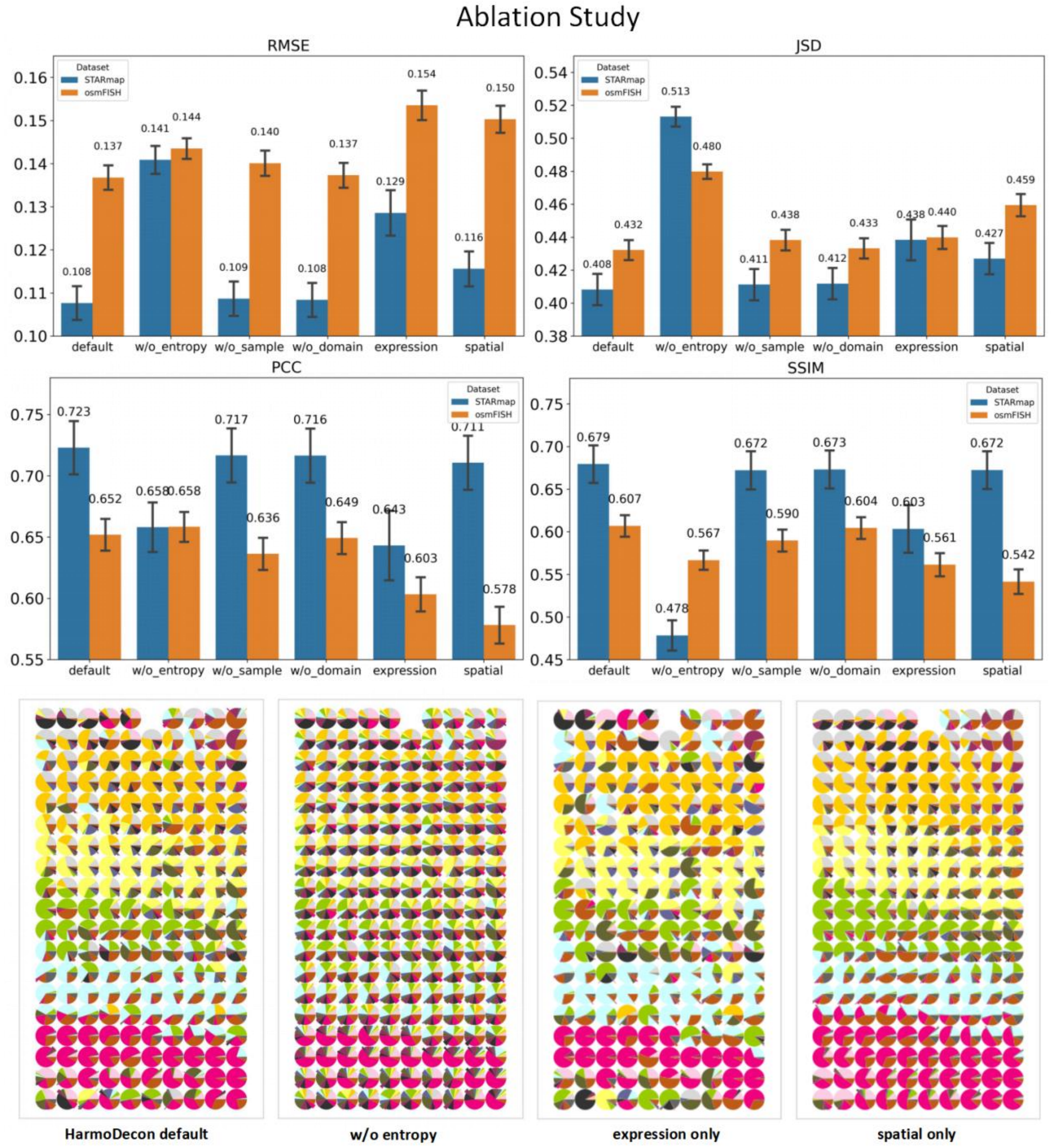

Supplementary Figure 15. Ablation study on loss items and the graph combination strategy of HarmoDecon. We assessed deconvolution performance by excluding one loss item and using a single graph across two benchmarking datasets (STARmap and osmFISH). We calculated four metrics, RMSE, JSD, PCC, and SSIM, compared to the ground truth. The default setting, which incorporates all loss items and utilizes both the expression graph and spatial graph, achieves superior overall performance. This suggests that the strategies we implemented effectively enhance deconvolution results.

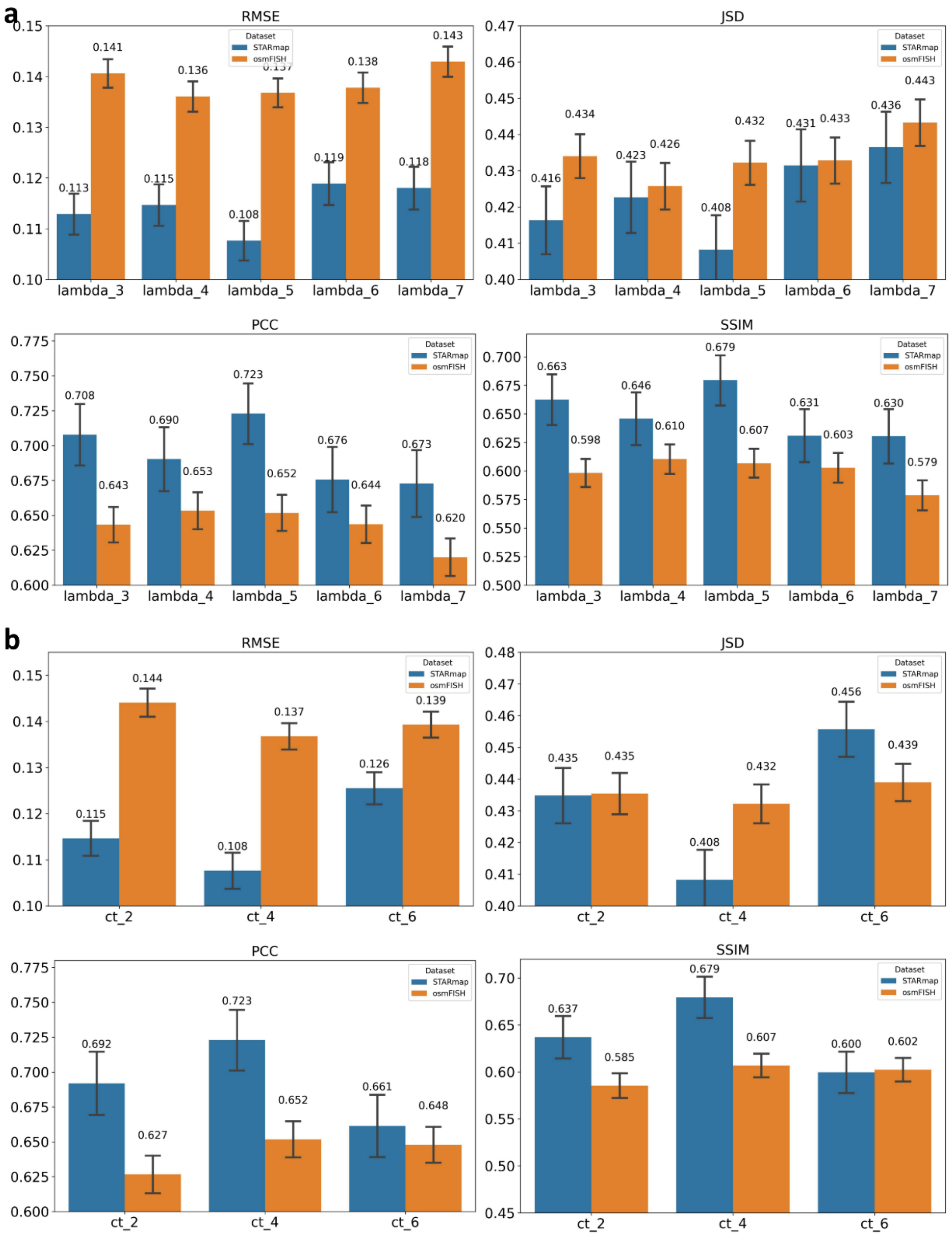
a
RMSE
Dataset
STARmap
osmFISH
0.113
0.141
0.115
0.136
0.108
0.119
0.138
0.118
0.143
lambda_3
lambda_4
lambda_5
lambda_6
lambda_7
JSD
0.416
0.434
0.423
0.426
0.408
0.432
0.431
0.433
0.436
0.443
PCC
0.708
0.643
0.690
0.653
0.723
0.652
0.676
0.644
0.673
0.620
SSIM
0.663
0.598
0.646
0.610
0.679
0.607
0.631
0.603
0.630
0.579
b
RMSE
0.115
0.144
0.108
0.137
0.126
0.139
ct_2
ct_4
ct_6
JSD
0.435
0.435
0.408
0.432
0.456
0.439
PCC
0.692
0.627
0.723
0.652
0.661
0.648
SSIM
0.637
0.585
0.679
0.607
0.600
0.602

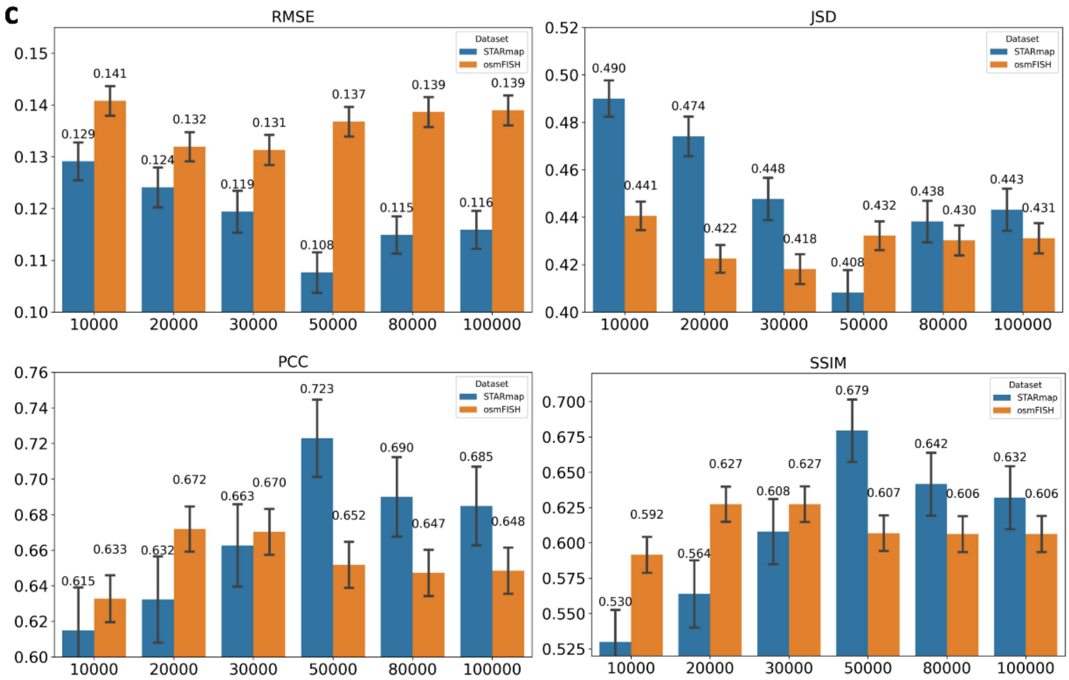

Supplementary Figure 16. Different settings during the pseudo-spots generation can influence the performance of HarmoDecon. We conducted a series of ablation experiments to recommend optimal hyperparameters that are effective across various scenarios. The findings indicated that the performance was optimal when the maximum number of cell types (Figure (a)), mean of Poisson distribution that control the number of cells (Figure (b)), and number of simulated spots (Figure (c)) were configured as 4, 5, and 50,000, respectively. We have selected the hyperparameters (4, 6, 50,000) as the default settings. When evaluating each parameter, the other parameters were maintained at their default values to ensure consistency and control variance.

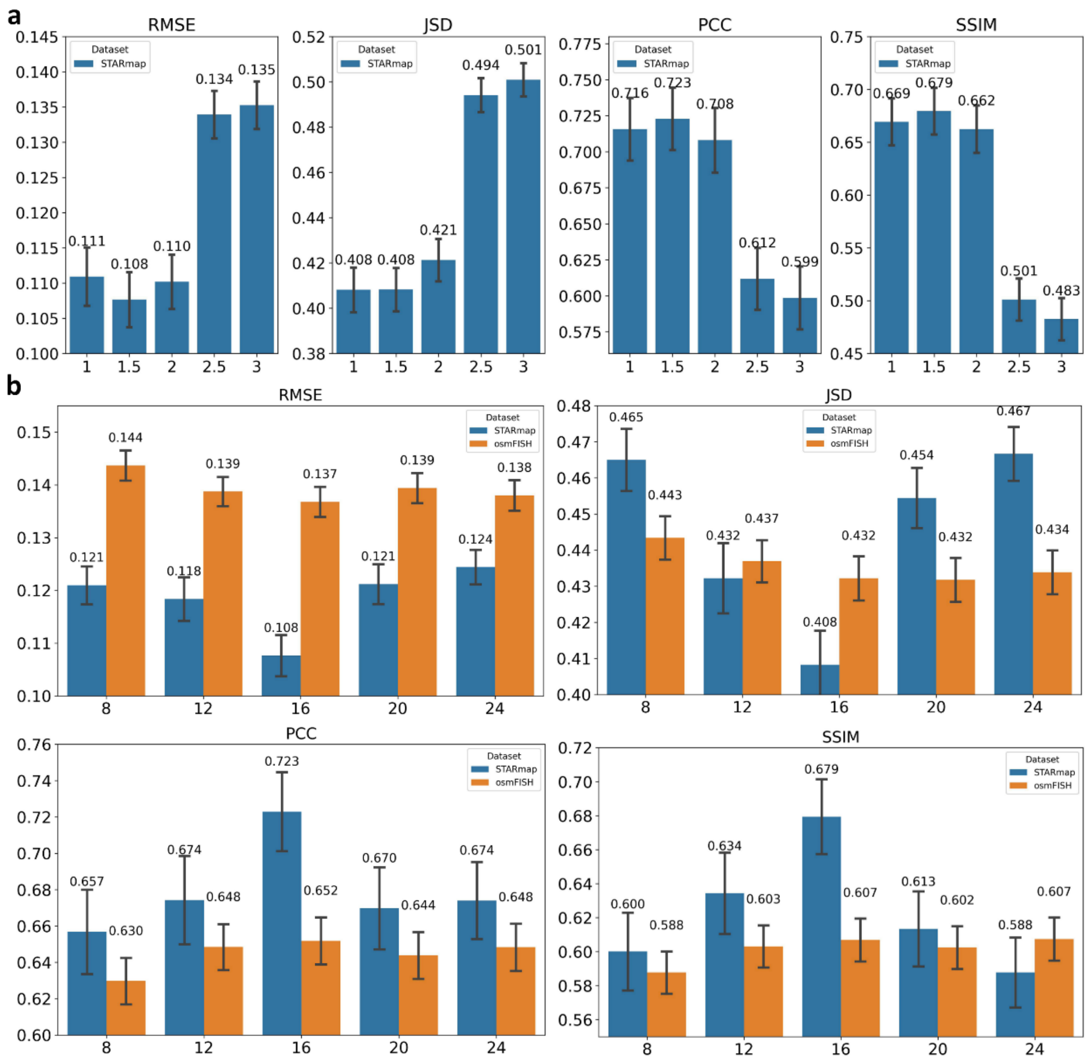

Supplementary Figure 17. Different settings during the training period can influence the performance of HarmoDecon. We conducted additional ablation experiments to thoroughly validate the strategies implemented during the training phase. Figure (a) illustrates that varying the Euclidean distance threshold for constructing the spatial graph has a substantial impact on performance. Notably, when the distance exceeds 2, there is a significant decrease in performance. (Note: The Euclidean distances calculated by coordinates [1, 1.5, 2, 2.5, 3] are approximately equivalent to K in K-nearest-neighbors [4, 8, 12, 20, 24]) in rectangular grids. Figure (b) shows that different selections of number of gaussian mixtures can affect the performance.

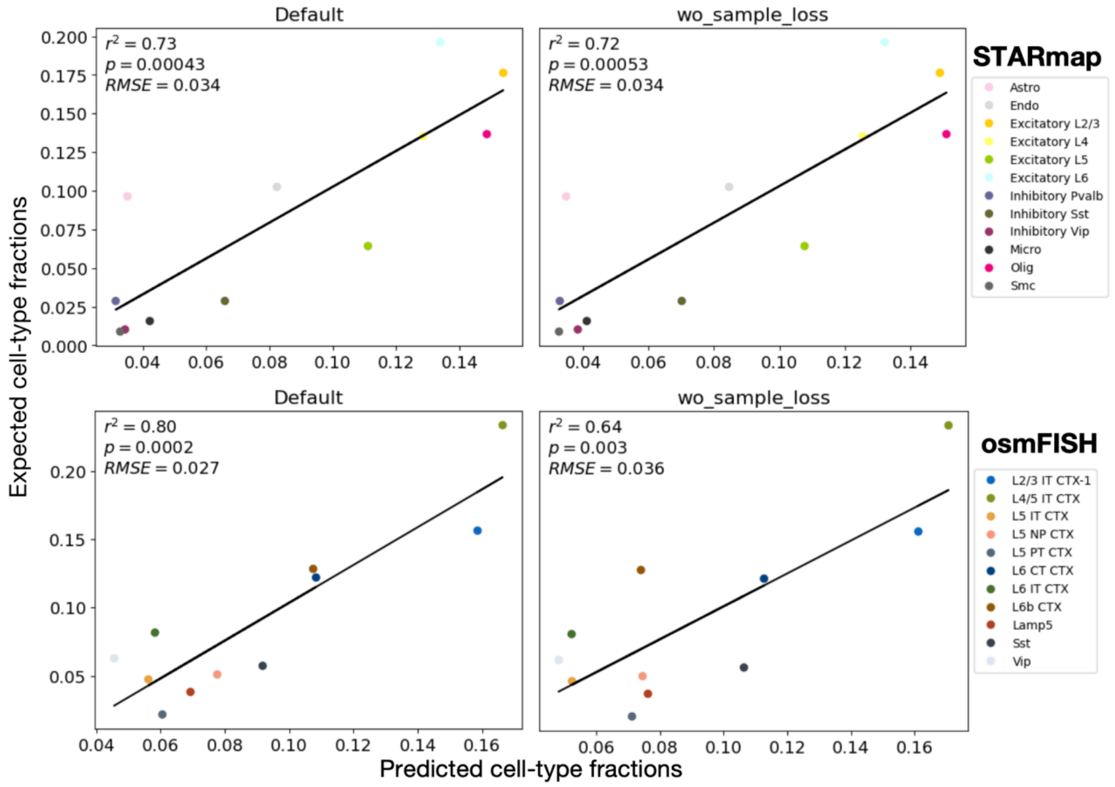

Supplementary Figure 18. The effect of sample loss on sample-level cell-type fractions. Left: HarmoDecon with default settings; Right: HarmoDecon without the sample loss. Upper: STARmap; Lower: osmFISH. The effect of sample loss is data-dependent. Compared to STARmap, the sample loss have more effect on the osmFISH data.

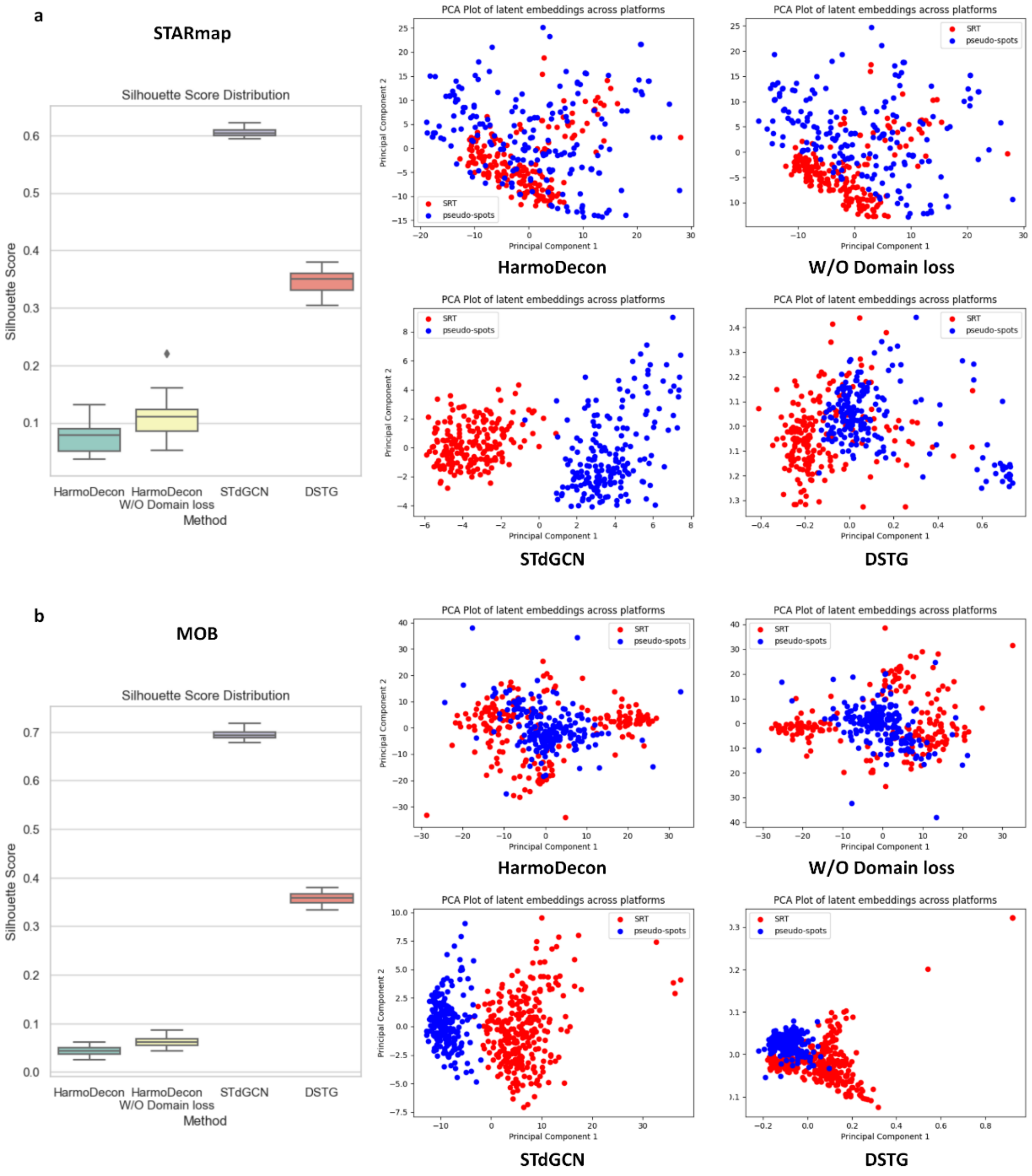

Supplementary Figure 19. Domain discriminator module aligns SRT and scRNA-seq data in a shared latent space. Visualization of the latent embeddings (before the final fully connected layer) for (a) STARmap and (b) MOB datasets, projected onto 2D PCA space. These datasets were selected due to their smaller number of spots, enabling clearer 2D visualization. HarmoDecon's domain discriminator module maps SRT spots and pseudo-spots (derived from scRNA-seq) into a harmonized latent space, where both domains mix seamlessly.

To quantify alignment, we computed the Silhouette Score—where a lower score indicates better domain mixing (unlike clustering tasks, where higher scores are desired). For each evaluation, 200 pseudo-spots were sampled, combined with SRT spots, and projected into 2D PCA space. The Silhouette Score was calculated between SRT and pseudo-spot groups, repeated 30 times, and summarized as a boxplot.

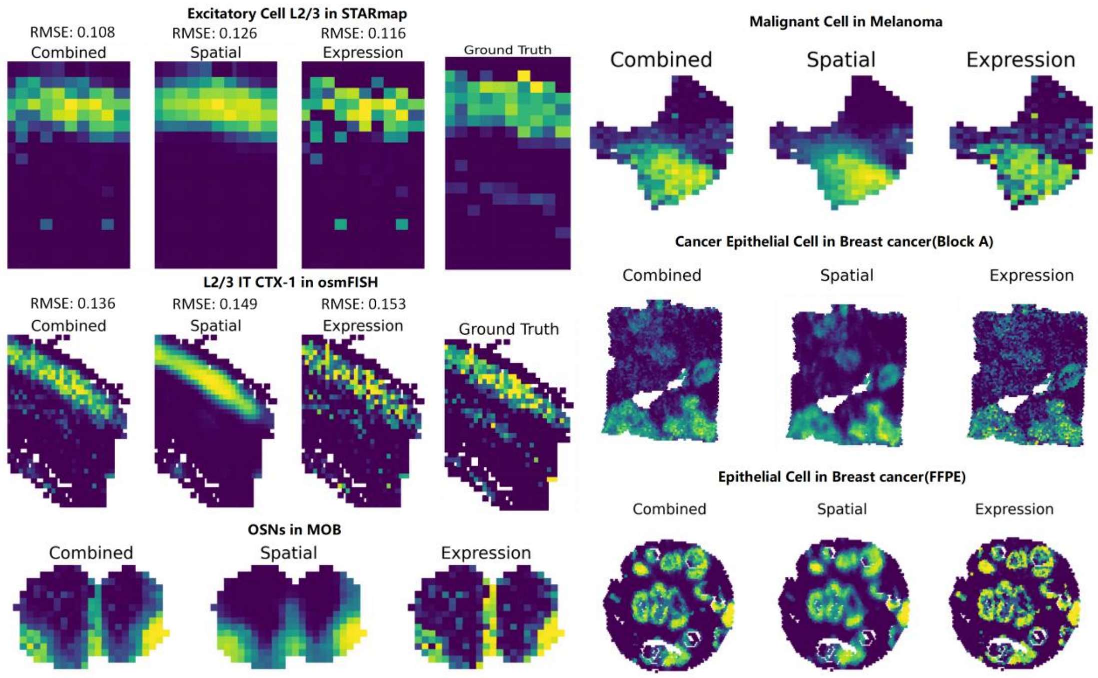

Supplementary Figure 20. HarmoDecon combines the results inferred by the spatial neighbor graph and the expression similarity graph In addition to gene expression profiles, HarmoDecon, being a graph-based model, accepts an extra graph as input. The edges within the graph are constructed based on either spatial neighbors or the similarity between the gene expression profiles of two spots. Across six real-world datasets, this combination consistently enhances performance. For instance, in single-cell spatial transcriptomics datasets like STARmap and osmFISH, the combined approach yields lower Root Mean Square Error (RMSE) compared to the ground truth. In bulk spatial transcriptomics datasets, the spatial component provides smoothness, while the expression component captures some domain-unseen features. For instance, in the OSNs (Olfactory Sensory Neurons) cells in the mouse olfactory bulb (MOB) dataset[6], the spatial component exhibits low false positives but may miss some border points, while the expression component contributes detailed information and detects missing values in the upper part of the olfactory nerve layer.

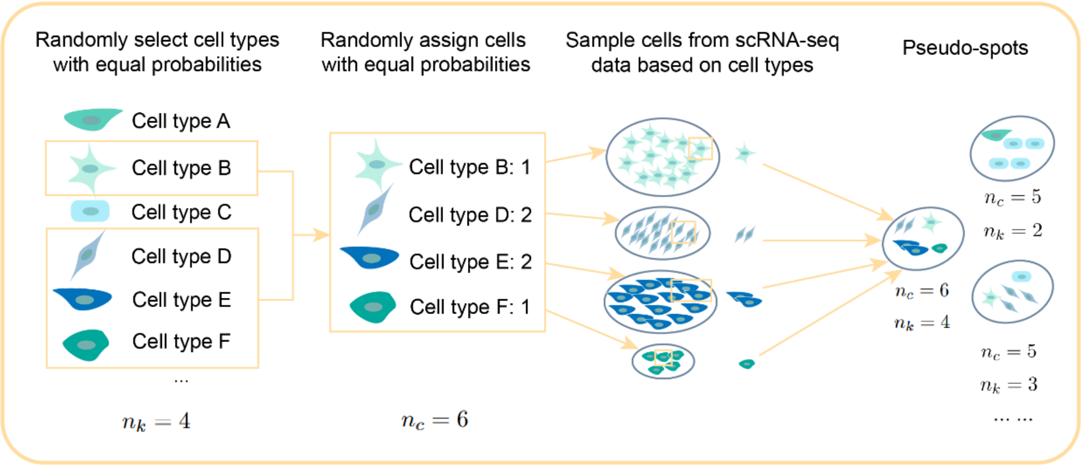

Supplementary Figure 21. Illustration of the pseudo spot generation process in HarmoDecon. After sampling values from a normal distribution (for the number of cell types) and Poisson distributions (for the number of cells), we describe the generation process for a spot with 4 cell types and 6 cells as follows: First, we randomly select 4 unique cell types from the entire cell type list, ensuring equal probabilities for each type. Next, we randomly assign 6 cells to the 4 selected cell types, again with equal probabilities. A cell type may receive zero cells or multiple cells. The gene expression profiles of the assigned cells are sampled from the corresponding cell pools, with only the selected cell types filtered from the original scRNA-seq data. Finally, we aggregate all the gene expression values of the sampled cells to create the gene expression profile of a pseudo spot, reflecting the known proportions of cell types.

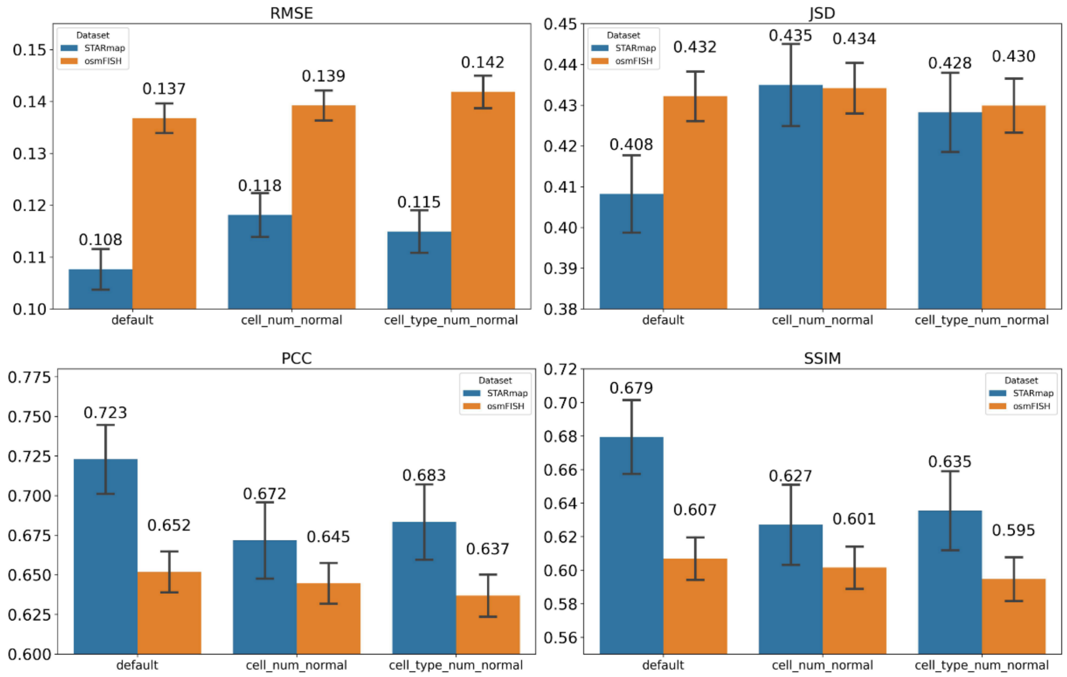

Supplementary Figure 22. Ablation study on the statistical distributions determining the number of cells and cell types. As described in the Methods section, when generating pseudo spots, we assume that the number of cells follows a Poisson distribution, while the number of cell types follows a uniform distribution. We validate the effectiveness of these distribution settings by comparing their performance against alternatives that utilize a Normal distribution (with the same mean value and a standard deviation set to 1) for sampling. Our results indicate that both the Poisson distribution for the number of cells and the uniform distribution for the number of cell types outperform their counterparts that use the Normal distribution. This finding suggests that, within the HarmoDecon framework, employing the default statistical distributions is more advantageous than using a Normal distribution.

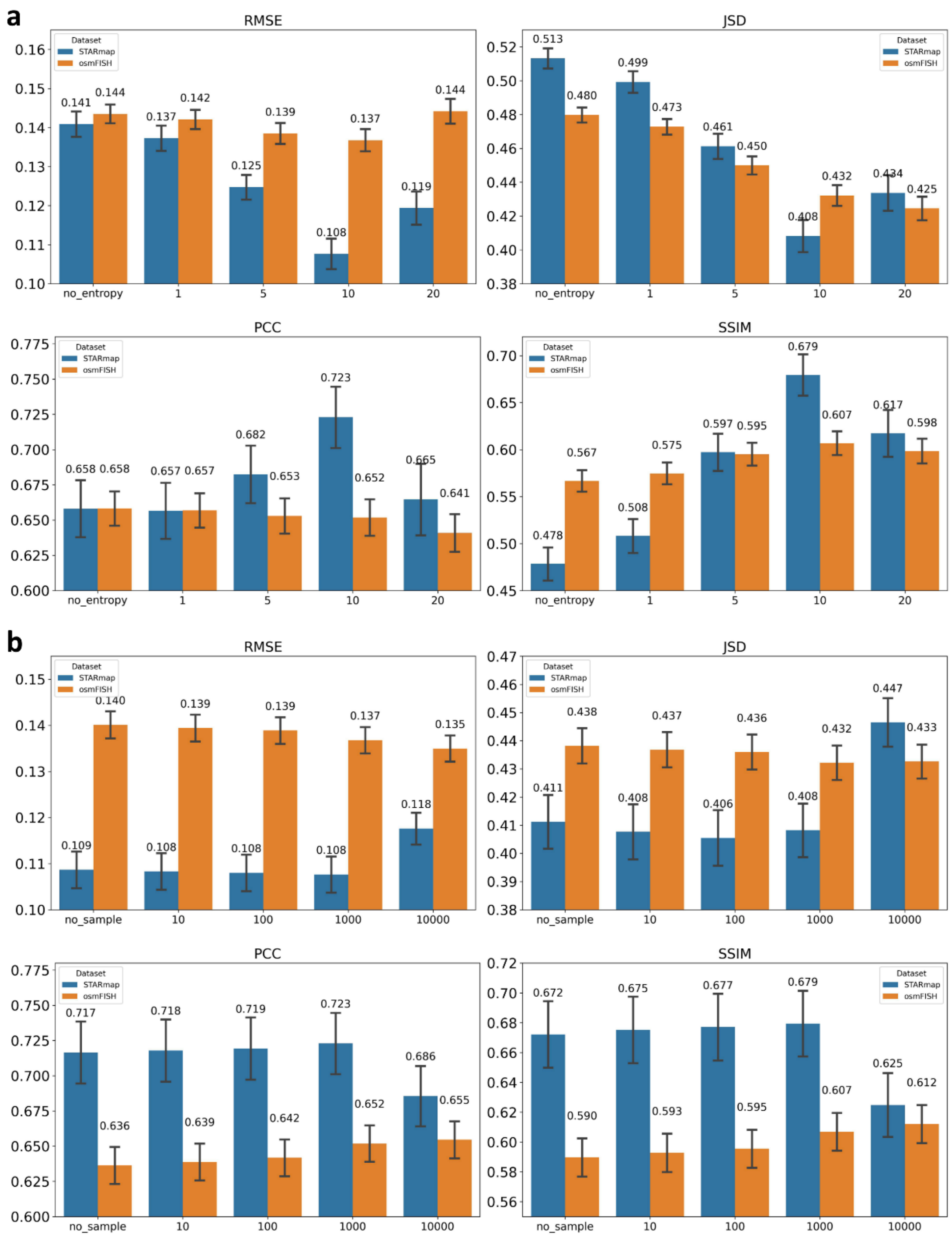

Supplementary Figure 23. Ablation study examining the effects of the entropy loss (a) and sample loss (b). While keeping other loss terms constant, we varied the weights of the entropy and sample losses to evaluate their impacts on model performance. We selected a weight of 10 for the entropy loss and 1000 for the sample loss, as these values yielded robust performance across the STARmap and osmFISH datasets. Notably, it was observed that excessively increasing the weight of a loss term could degrade performance. For instance, when the sample loss weight was set to 10,000, the model's performance deteriorated compared to the baseline model without the sample loss, suggesting that disproportionately scaling a single loss component may adversely affect overall results.

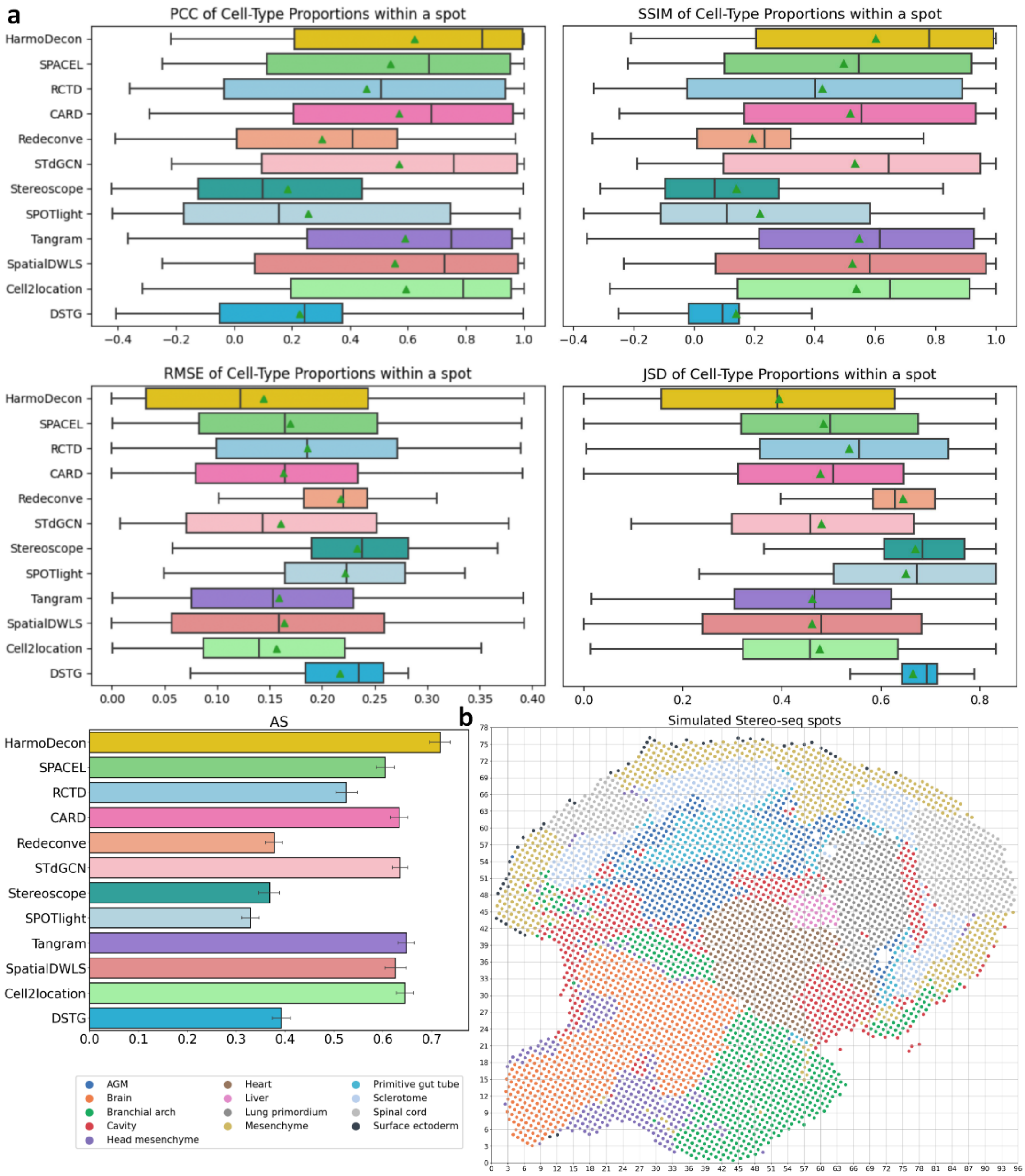

Supplementary Figure 24. Evaluation on simulated multi-cell spots from MOSTA Stereo-seq[8]. (a) Boxplots comparing four metrics (PCC, SSIM, RMSE, and JSD) across methods, evaluated on n = 611 spots simulated by gridding SRT data. Our method consistently outperforms existing approaches across all metrics while also achieving superior accuracy scores. (b) Schematic of the simulation workflow: each multi-cell spot aggregates transcriptomes from 1 to 10 cells. The MOSTA dataset comprises mouse embryo sections with comprehensive cell-type annotations spanning diverse organs and tissues.

We use the annotation from E9.5_E2S1.MOSTA.h5ad as the single cell reference, and use E9.5_E2S3.MOSTA.h5ad, which has the most similar cell type components to slice E2S1, is used as the estimated SRT for deconvolution.

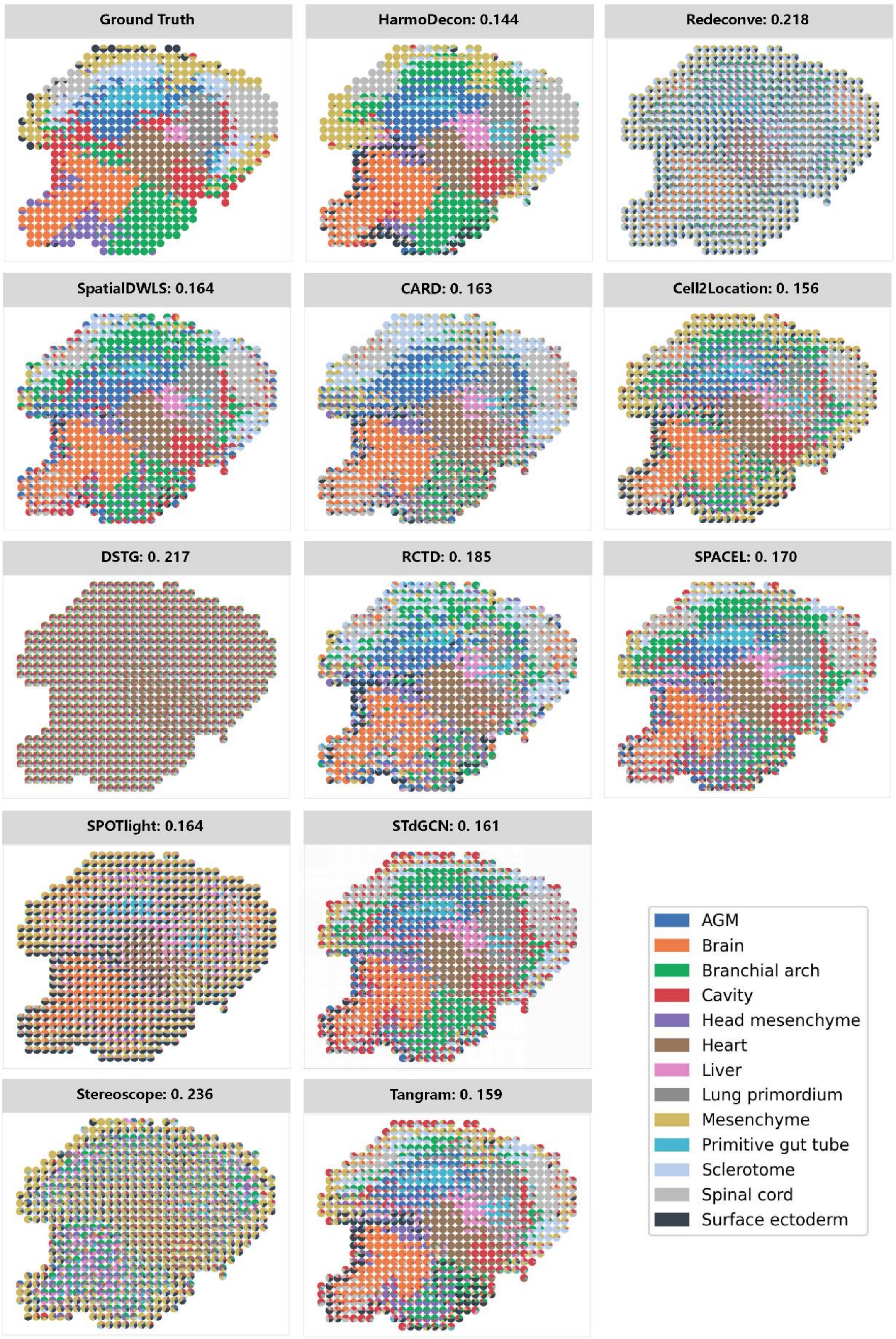

Supplementary Figure 25. Pie chart of the deconvolution results of Stereo-seq mouse embryo data. Quantitative comparison of RMSE values, with HarmoDecon achieving the lowest error (annotated above each panel). Results highlight our method's statistically significant improvement in accuracy over existing approaches for embryonic development analysis.

Average Cell type proportions in the granule cell layer (GCL)

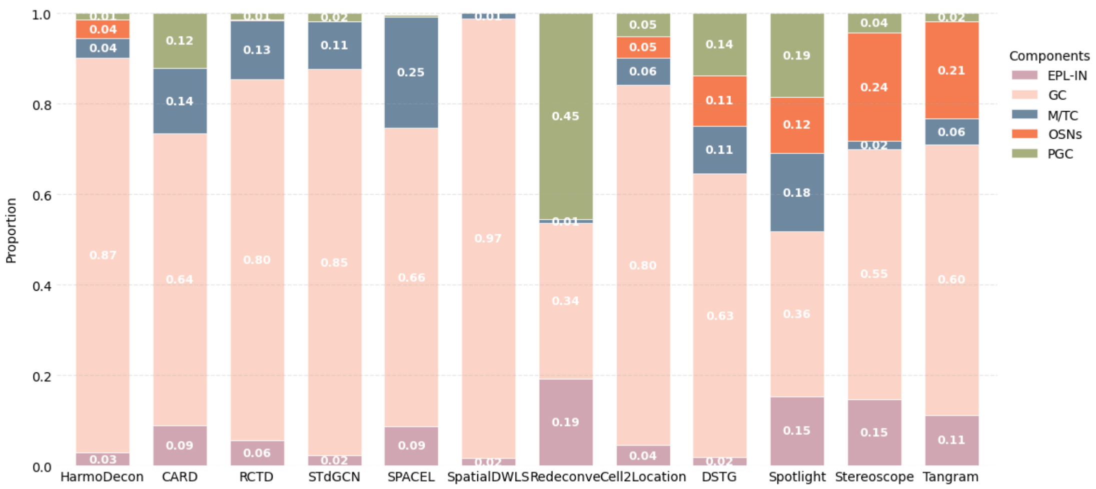

Average Cell type proportions in the mitral cell layer (MCL)

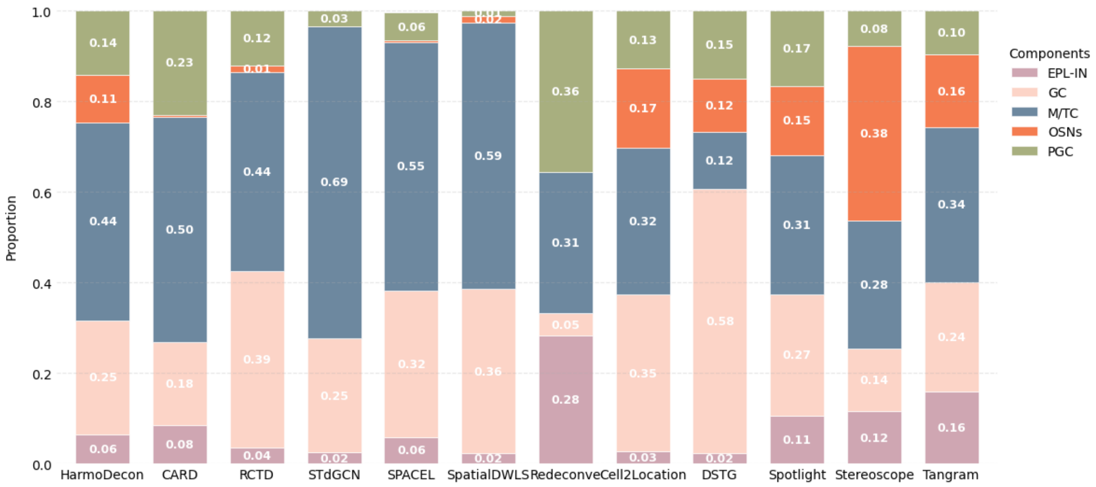

Average Cell type proportions in the glomerular layer (GL)

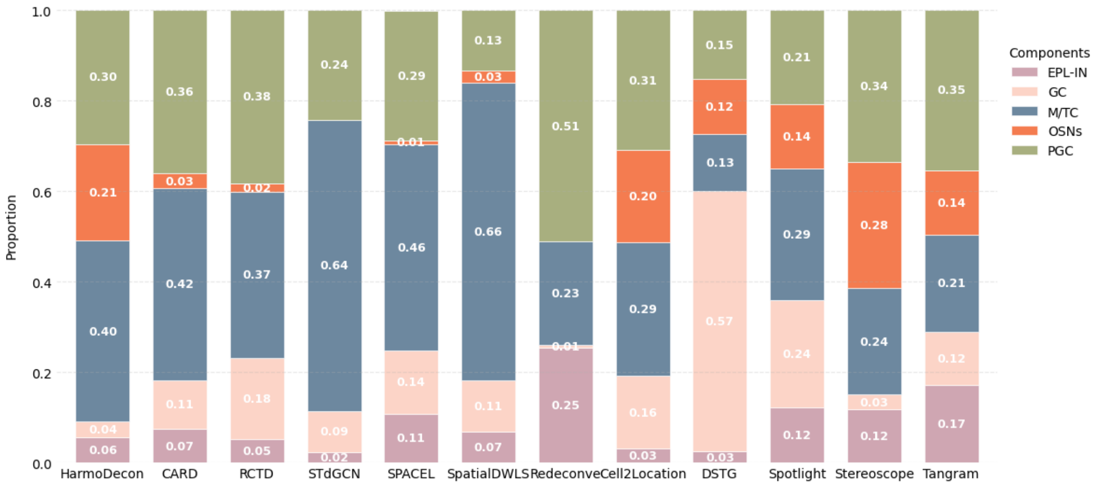

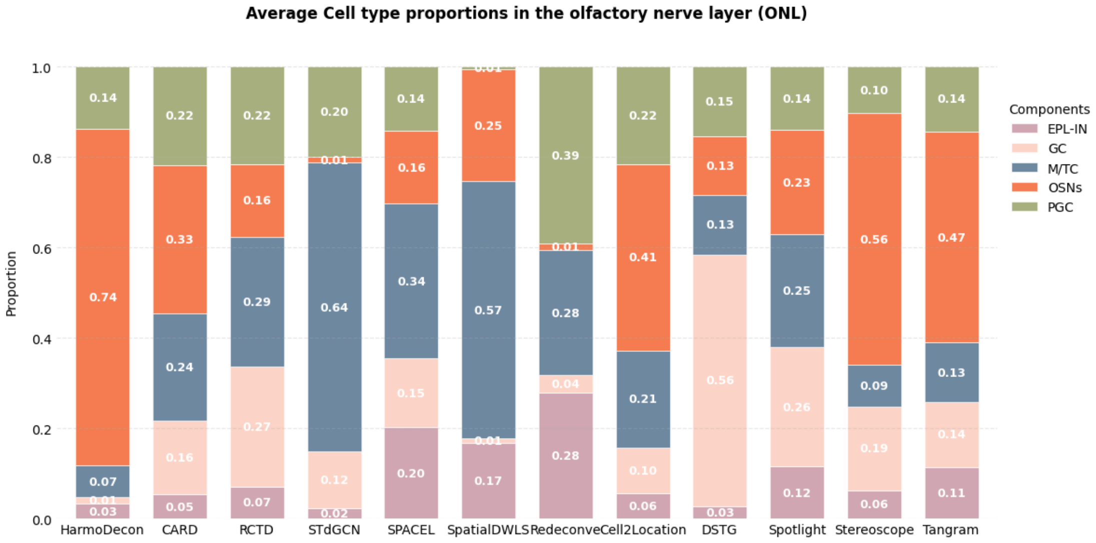

Supplementary Figure 26. Average cell type proportions across the four domains in the Mouse Olfactory Bulb (MOB) dataset. Compared to other comparison methods, only HarmoDecon well captures the enrichment of both granule cells (GC) in the granule cell layer (GCL) and olfactory sensory neurons (OSNs) in the olfactory nerve layer (ONL).

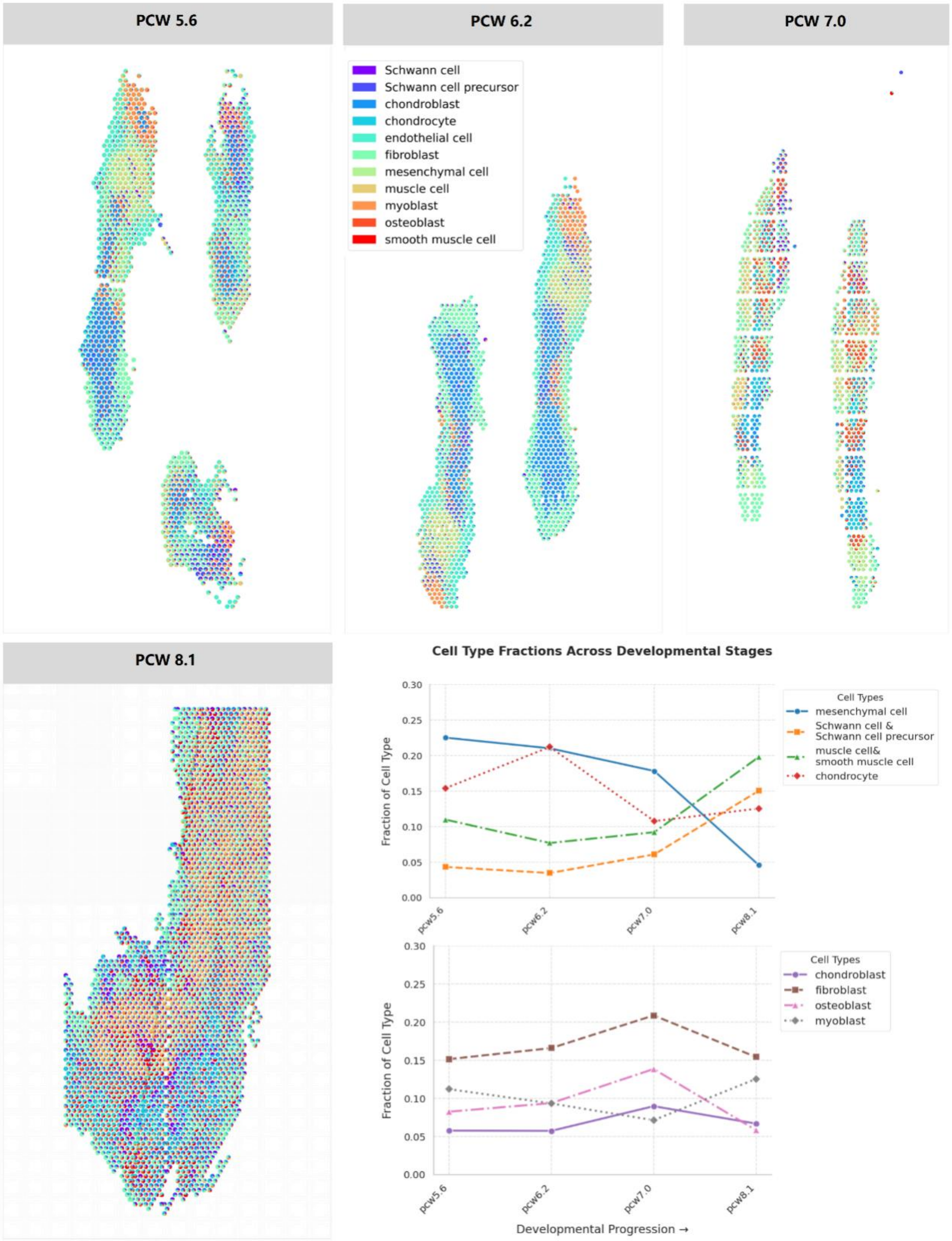

Supplementary Figure 27. Experiments on 10x Visium developing human limbs dataset[9] demonstrate the importance of sample-level cell-type fraction. We downloaded the developing human limbs SRT and paired single-cell RNA-seq data from the cellxgene website. By observing the sample-level cell-type fractions across developmental stages, we find that during the developmental progression, undifferentiated cells, like mesenchymal cells decrease. While more differentiated cells, like Schwann cells and muscle cells increase. The fraction of cells in the middle of differentiation , like various kinds of blast cells, showed a fluctuating trend.

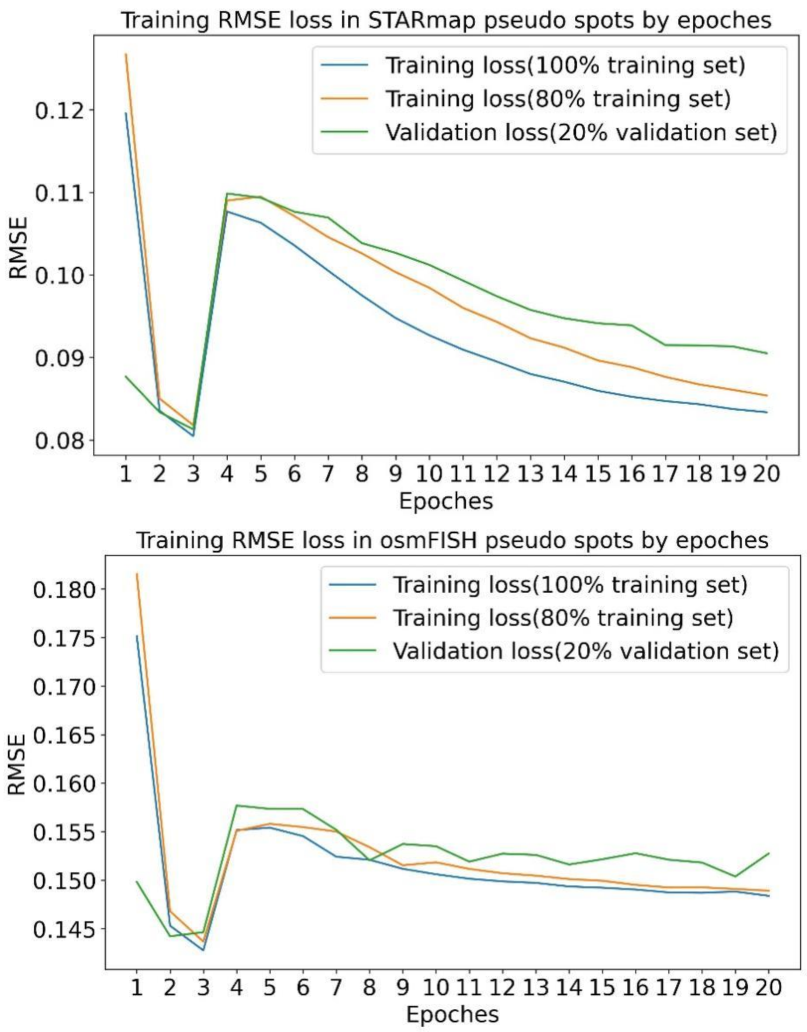

Supplementary Figure 28. Evaluation of training loss curves for the osmFISH and STARmap datasets using an early stopping strategy. By default, HarmoDecon does not employ early stopping and trains all datasets for 20 epochs. The blue line represents the training loss curve using 50,000 pseudo-spots (default setting in HarmoDecon). The orange and yellow lines depict the training and validation losses, respectively, with an 8:2 split (40,000 pseudo-spots for training, 10,000 for validation). An optional early stopping function is provided in the source code for users to customize training on their own data.

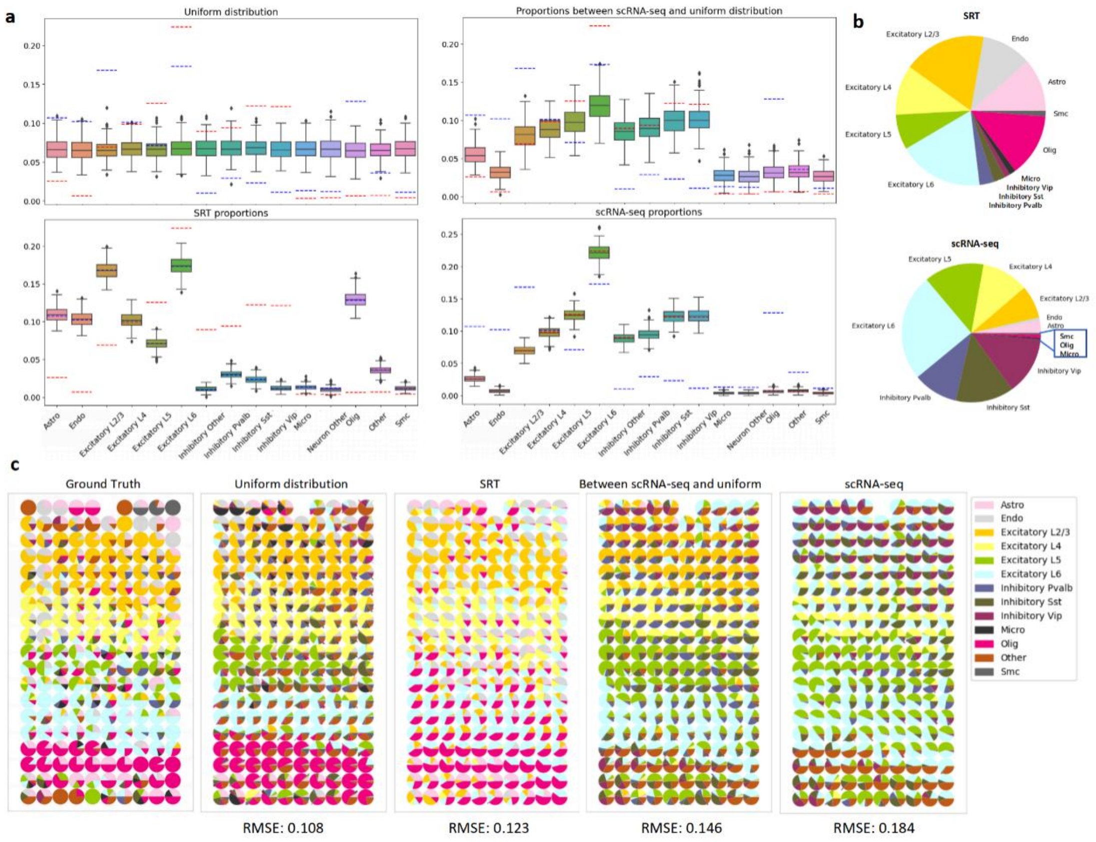

Supplementary Figure 29 Evaluation of sampling strategies for pseudo-spot generation and their impact on deconvolution performance in the STARmap dataset.

(a) Boxplots showing cell-type distributions of pseudo-spot graphs generated under four different sampling ratios (n=250 graphs per setting, each containing 200 pseudo-spots). Cell-type proportions were averaged across pseudo-spots within each graph. Graphs (rather than individual spots) are plotted as they better represent the "sample" level. Red dotted lines indicate scRNA-seq-derived cell-type proportions, while blue dotted lines show the true spatial transcriptomics (SRT) proportions (included here for ablation analysis only; these data should normally be unseen at this stage).

(b) Pie charts comparing cell-type distributions between scRNA-seq and SRT data in the STARmap dataset.

(c) Deconvolution results (pie charts) from pseudo-spots generated using the four sampling strategies.

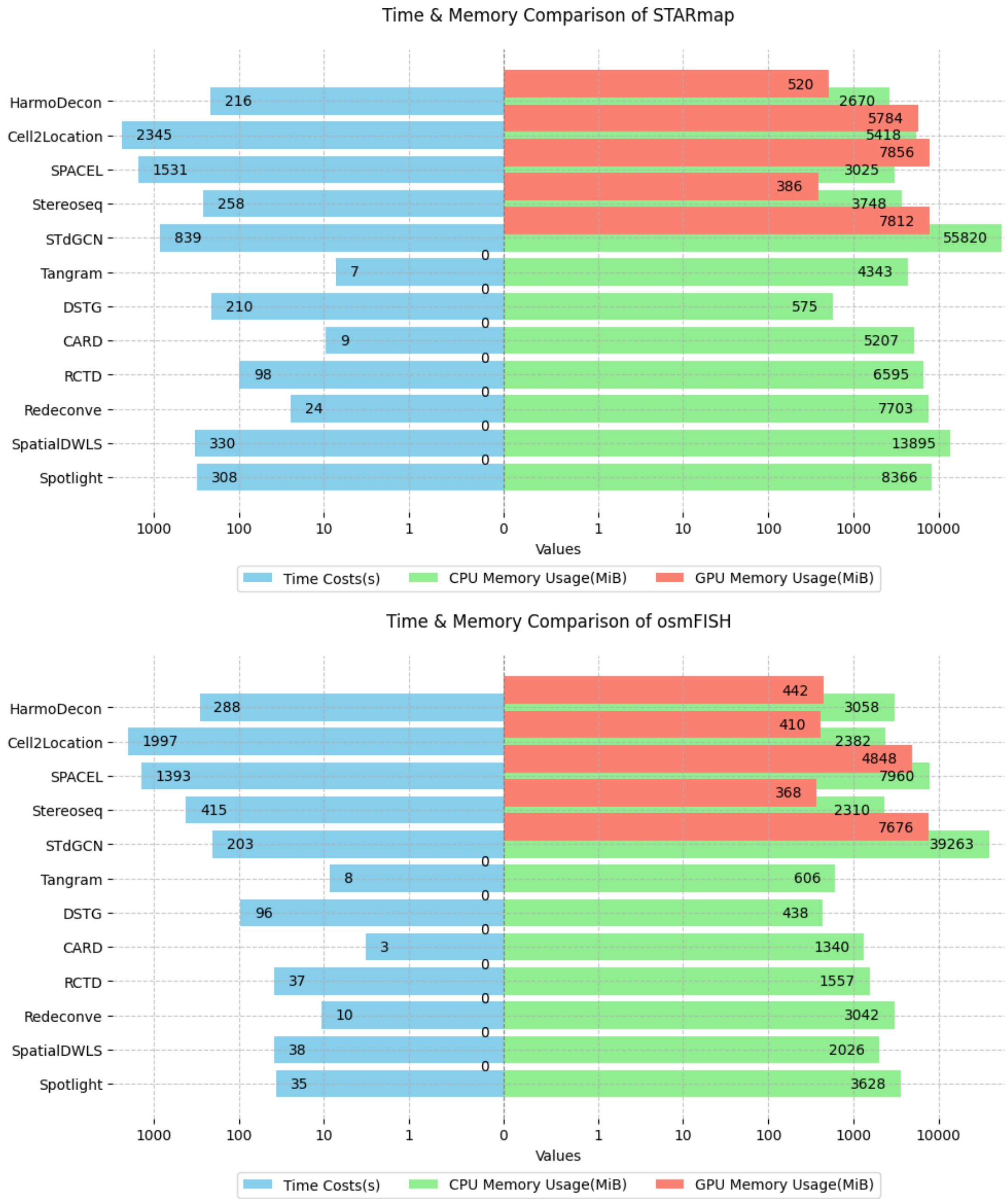

Supplementary Figure 30 The runtime and memory consumption of HarmoDecon and Compared methods in the STARmap[1] and osmFISH data[3].

Reference

1. Xiao Wang et al. ,Three-dimensional intact-tissue sequencing of single-cell transcriptional states.Science361,eaat5691(2018). doi: 10.1126/science.aat5691
2. Li, B., Zhang, W., Guo, C. et al. Benchmarking spatial and single-cell transcriptomics integration methods for transcript distribution prediction and cell type deconvolution. Nat Methods 19, 662–670 (2022). doi: 10.1038/s41592-022-01480-9
3. Codeluppi, S., Borm, L.E., Zeisel, A. et al. Spatial organization of the somatosensory cortex revealed by osmFISH. Nat Methods 15, 932–935 (2018). doi: 10.1038/s41592-018-0175-z
4. Chen, J., Liu, W., Luo, T., Yu, Z., Jiang, M., Wen, J., Gupta, G. P., Giusti, P., Zhu, H., Yang, Y., & Li, Y. (2022). A comprehensive comparison on cell-type composition inference for spatial transcriptomics data. Briefings in bioinformatics, 23(4), bbac245. doi: 10.1093/bib/bbac245
5. 10x Genomics. Visium Spatial Gene Expression. 10x Genomics website (2020).
6. Ståhl, P. L., Salmén, F., Vickovic, S., Lundmark, A., Navarro, J. F., Magnusson, J., Giacomello, S., Asp, M., Westholm, J. O., Huss, M., Mollbrink, A., Linnarsson, S., Codeluppi, S., Borg, Å., Pontén, F., Costea, P. I., Sahlén, P., Mulder, J., Bergmann, O., Lundeberg, J., … Frisén, J. (2016). Visualization and analysis of gene expression in tissue sections by spatial transcriptomics. Science (New York, N.Y.), 353(6294), 78–82. doi: 10.1126/science.aaf2403
7. Thrane, K., Eriksson, H., Maaskola, J., Hansson, J. & Lundeberg, J. Spatially resolved transcriptomics enables dissection of genetic heterogeneity in stage iii cutaneous malignant melanoma. Cancer Research 78, 5970–5979 (2018).
8. Chen, A., Liao, S., Cheng, M., Ma, K., Wu, L., Lai, Y., ... & Wang, J. (2022). Spatiotemporal transcriptomic atlas of mouse organogenesis using DNA nanoball-patterned arrays. Cell, 185(10), 1777-1792. DOI: 10.1016/j.cell.2022.04.003
9. Zhang, B., He, P., Lawrence, J.E.G. et al. A human embryonic limb cell atlas resolved in space and time. Nature 635, 668–678 (2024). https://doi.org/10.1038/s41586-023-06806-x
10. Vahid, M.R., Brown, E.L., Steen, C.B. et al. High-resolution alignment of single-cell and spatial transcriptomes with CytoSPACE. Nat Biotechnol 41, 1543–1548 (2023). https://doi.org/10.1038/s41587-023-01697-9
11. Wu, S.Z., Al-Eryani, G., Roden, D.L. et al. A single-cell and spatially resolved atlas of human breast cancers. Nat Genet 53, 1334–1347 (2021). https://doi.org/10.1038/s41588-021-00911-1
